# Taxonomic variation, plastic degradation, and antibiotic resistance traits of plastisphere communities in the maturation pond of a wastewater treatment plant

**DOI:** 10.1128/aem.00715-24

**Published:** 2024-09-27

**Authors:** Stefan D. M. Maday, Joanne M. Kingsbury, Louise Weaver, Olga Pantos, Jessica A. Wallbank, Fraser Doake, Hayden Masterton, Maisie Hopkins, Rosa Dunlop, Sally Gaw, Beatrix Theobald, Regis Risani, Robert Abbel, Dawn Smith, Kim M. Handley, Gavin Lear

**Affiliations:** 1 School of Biological Sciences, University of Auckland, Auckland, New Zealand; 2 Institute of Environmental Science and Research, Christchurch, New Zealand; 3 School of Physical and Chemical Sciences, University of Canterbury, Christchurch, New Zealand; 4 Scion, Rotorua, New Zealand; Norwegian University of Life Sciences, Ås, Norway

**Keywords:** antimicrobial resistance, microplastics, plasticDB, 16S rRNA gene, shotgun metagenomics

## Abstract

Wastewater treatment facilities can filter out some plastics before they reach the open environment, yet microplastics often persist throughout these systems. As they age, microplastics in wastewater may both leach and sorb pollutants and fragment to provide an increased surface area for bacterial attachment and conjugation, possibly impacting antimicrobial resistance (AMR) traits. Despite this, little is known about the effects of persistent plastic pollution on microbial functioning. To address this knowledge gap, we deployed five different artificially weathered plastic types and a glass control into the final maturation pond of a municipal wastewater treatment plant in Ōtautahi-Christchurch, Aotearoa/New Zealand. We sampled the plastic-associated biofilms (plastisphere) at 2, 6, 26, and 52 weeks, along with the ambient pond water, at three different depths (20, 40, and 60 cm from the pond water surface). We investigated the changes in plastisphere microbial diversity and functional potential through metagenomic sequencing. Bacterial 16S ribosomal RNA genes composition did not vary among plastic types and glass controls (*P* = 0.997) but varied among sampling times [permutational multivariate analysis of variance (PERMANOVA), *P* = 0.001] and depths (PERMANOVA, *P* = 0.011). Overall, there was no polymer-substrate specificity evident in the total composition of genes (PERMANOVA, *P* = 0.67), but sampling time (PERMANOVA, *P* = 0.002) and depth were significant factors (PERMANOVA, *P* = 0.001). The plastisphere housed diverse AMR gene families, potentially influenced by biofilm-meditated conjugation. The plastisphere also harbored an increased abundance of genes associated with the biodegradation of nylon, or nylon-associated substances, including nylon oligomer-degrading enzymes and hydrolases.

**IMPORTANCE:**

Plastic pollution is pervasive and ubiquitous. Occurrences of plastics causing entanglement or ingestion, the leaching of toxic additives and persistent organic pollutants from environmental plastics, and their consequences for marine macrofauna are widely reported. However, little is known about the effects of persistent plastic pollution on microbial functioning. Shotgun metagenomics sequencing provides us with the necessary tools to examine broad-scale community functioning to further investigate how plastics influence microbial communities. This study provides insight into the functional consequence of continued exposure to waste plastic by comparing the prokaryotic functional potential of biofilms on five types of plastic [linear low-density polyethylene (LLDPE), nylon-6, polyethylene terephthalate, polylactic acid, and oxygen-degradable LLDPE], glass, and ambient pond water over 12 months and at different depths (20, 40, and 60 cm) within a tertiary maturation pond of a municipal wastewater treatment plant.

## INTRODUCTION

Recently, the pervasive and ubiquitous distribution of plastic pollution has been extensively studied for its diverse impacts on marine biota and chemistry. Studies have documented the frequency and abundance of plastic pollution, including primary and secondary micro- and nano-plastics (1–3). Occurrences of plastics causing entanglement or ingestion (4, 5) and the leaching of toxic additives and persistent organic pollutants from environmental plastics and their consequences for marine macrofauna are widely reported (6–9). Less well-studied is the effect of plastic pollution on microorganisms, which provide essential ecosystem services. Most studies exploring plastic impacts on microorganisms have focused on changes in community composition and only infer impacts on community functioning (10–12). However, in the last five years, a handful of studies have begun exploring the functional potential of the plastic-associated microbiome, or the “plastisphere,” through metagenomic sequencing, aided by recent advances in culture-independent and computational tools. Previous studies have characterized diverse plastisphere functional traits, including the impacts plastics have on photosynthesis (13), the spread of antimicrobial resistance (AMR) (14–16), and polymer degradation traits (17); yet, a large knowledge gap remains in terms of how these plastisphere communities vary among different plastic types, particularly in environments with high anthropogenic impact.

Municipal wastewater treatment plants (WWTP) provide unique opportunities for studying the impacts of plastic pollution on microbial communities since they are typically enriched with microbial life, including pathogens (18), and diverse waste substances, including plastics, but also antibiotics and persistent emerging contaminants (19, 20). Globally, most municipal WWTPs follow a similar design: larger particulates are removed with a screen mesh, normally >6 mm, before secondary treatment removes suspended and dissolved organic nutrients. Pathogenic microbes may be removed during secondary treatment through UV sunlight disinfection and high pH (driven through algal photosynthesis) before the water is passed through tertiary treatment ponds to improve water quality further. While WWTPs remove the majority of large plastic particles (21), particles <5 mm may persist in the treated material that is released into the environment (22, 23). Notably, aging plastics sorb organic pollutants, including antibiotics (24), but also trace elements and pathogens, while leaching chemical additives such as stabilizers, flame retardants, antioxidants, and plasticizers (25) into the environment, impacting WWTP effluent microbiota (13, 26, 27). Thus, persistent microplastics may impact microbiota functionality in WWTPs and their receiving waters.

Plastics in WWTPs may influence microbial diversity and functionality by (1) providing a platform to facilitate conjugation (28, 29) and the dissemination of AMR genes (30) and (2) providing a more prolonged exposure of their associated communities to antibiotics, additives, and other contaminants, which may further increase rates and extents of microbial conjugation (31). Several studies have found pathogenic and antimicrobial-resistant microbes at higher concentrations within plastisphere communities than in the surrounding environment (32–34). However, the strength of this relationship for plastisphere communities associated with wastewater remains unclear. Because antibiotics from farming and human populations can pass through wastewater treatment systems, their elevated concentrations may further influence microbial diversity and function. Additional investigation is necessary to understand the functional potential of plastic-associated biofilms in WWTPs, including in relation to the abundance of potential pathogens and antimicrobial-resistant organisms.

Studies exploring the impact of plastic pollution on microbial communities have generally focused on their taxonomic features (10, 12, 35), with few studies yet exploring their broader genetic traits. Microbial communities that differ in their taxonomic compositions may show equivalent functional potentials due to functional redundancy (36, 37). Alternatively, those that show little taxonomic difference might nevertheless demonstrate substantial variation in their transcriptional or translational activity. It is, therefore, important to consider the functional attributes and response of biofilms to plastic pollution. Indeed, studies have demonstrated that microbial enzyme activity is significantly altered following exposure to plastics (38, 39), including the increased expression of predicted plastic biodegradation genes and enzymes (40–42). Detailed knowledge of the functional potential of entire biofilms exposed to diverse plastic substrates is currently lacking, particularly in WWTP environments. Therefore, by studying the genetic inventory of WWTP biofilms, we can understand the potential effects plastics have on wastewater microbiota and possible insights into related bioremediation processes.

Exploring functional and compositional changes in WWTP plastisphere communities over time will provide insight into how they adapt to the provision of novel plastic substrates. We compared the prokaryotic functional potential of biofilms on five types of plastic [linear low-density polyethylene (LLDPE), nylon-6 (PA), polyethylene terephthalate (PET), polylactic acid (PLA), oxygen-degradable LLDPE (oxo-LLDPE)], glass, and ambient pond water over 12 months and at different depths (20, 40, and 60 cm) within a tertiary maturation pond. Specifically, we investigated changes in (1) biofilm taxonomic community composition, (2) broad-scale functional potential using shotgun metagenomic methods, (3) the presence of putative plastic-degrading genes, and (4) antimicrobial resistance mechanisms within the biofilm.

## MATERIALS AND METHODS

### Sampling site and collection

**Fig 1:**
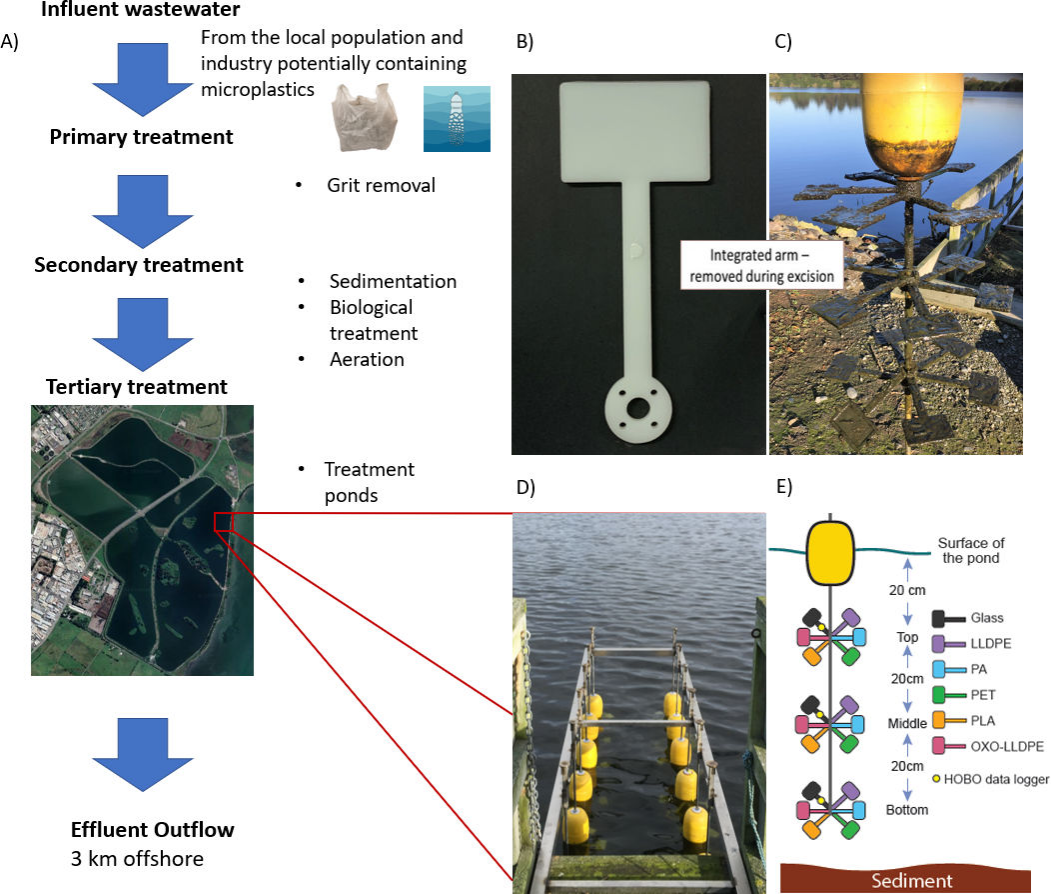
Study site schematic and design: (**A**) flow diagram outlining the treatment process at the Ōtautahi-Christchurch WWTP, also showing the approximate location of the deployment site near the effluent outflow, as highlighted by the red box; (**B**) paddle design; (**C**) a single pole of plastic paddles (sampled 6 weeks after sample deployment); (**D**) the deployment structure viewed from above; and (**E**) a schematic of the deployment structure, including the orientation and the placement distances of samples under the water.

Plastics were installed in the final maturation pond of a wastewater treatment plant in Ōtautahi-Christchurch, Aotearoa/New Zealand (NZ), at a latitude of 43°31’55.2’’S and longitude of 172°43’18.8’’E. The site is the largest WWTP in Te Waipounamu, South Island of NZ. The influent water undergoes an initial screening (10 mm) to remove grit, followed by primary sedimentation, biological treatment, aeration, and clarification (23) (Fig. 1). The site has an average influent flow rate of 172,000,000 L daily (43), serving Christchurch urban and Tai Tapu (combined population of ~385,000). A series of six maturation ponds receive secondary treated effluent from the treatment plant. The wastewater flows under gravity through these ponds, with an estimated travel time (residence time) of 25–30 days, before being discharged 3 km offshore into the Pacific Ocean. The primary objective of the series of maturation ponds at this WWTP is to reduce biochemical oxygen demand/chemical oxygen demand via further organic matter removal and for disinfection to occur through UV sunlight inactivation. A previous study calculated that microplastics in these maturation ponds ranged from 0.7 to 1.9 particles/L (23).

As per Wallbank et al. (10), plastic paddles for biofilm sampling were installed at three heights (20 cm, 40 cm and 60 cm below the water surface) on stainless-steel (316) poles using a stainless-steel (316) frame attached to a fixed jetty. Plastic paddles for chemical analysis were similarly attached. The jetty was located in the final maturation pond 50–100 m south of the proximal outflow (Fig. 1). Buoys on each pole allowed for samples to remain at a constant depth despite changes in pond depth due to varying flow rates and rainfall. Data loggers (HOBO Pendant MX2202; Onset Computer Corp Bourne, MA, USA) were attached to record temperature and light intensities at the three depths, recording every 15 minutes throughout the experiment. The average water temperature surrounding the deployment structure ranged between 8.64°C and 19.17°C (Table S1).

The base polymer types used in this study were chosen due to their high commercial use, abundance as marine contaminants (23, 44, 45), and their varied degradation potential (46, 47). These plastics consisted of LLDPE, oxo-LLDPE, polyamide-6 (also known as nylon-6; PA), PET, and PLA. Plastics contained additives typically included for reduced UV/light degradation, except for oxo-LLDPE, which contained catalytic manganese stearate (0.2%) for increased oxidation of the base polymer (11) (Table S2). Before deployment, all plastics were assessed via thermogravimetric analysis (TGA) to quantify their inorganic content. Inorganic additive contents were <0.5% for all study plastics (Table S3) (11). All other additives were below detection limits (±0.1% of the original mass) (Table S2). All plastics were injection molded into rectangular paddles (76 × 51 × 4 mm) with an integrated arm (10, 11, 48) (Fig. 1B) and artificially weathered using a modified accelerated weathering (QUV) tester (ASTM D4329-13/ASTM G154; ASTM International, 2023). The weathering protocol ran for 800 hours (400 hours per paddle side), repeating cycles of 8 hours of UVA-340 (preferred irradiance at 340 nm was 0.89 W/m^2^) UV light at 50°C [reduced from 60°C due to the low glass transition temperature (Tg) of PLA] and 4 hours of condensation (to mimic dew) at 50°C. Paddle holders were moved across the accelerated weathering (QUV) tester once weekly to minimize exposure variation at the edges. Additionally, sterile glass slides (76 × 51 × 1 mm) were used as experimental control replicates, made from white low-iron soda-lime float-glass (ProSciTech, Australia). Plastic paddles were fixed to the stainless-steel poles via an integrated arm; glass slides were fixed to the structure using stainless steel frames (Fig. 1C through E). An additional set of dog bone-shaped paddles were similarly installed, with dimensions in agreement with the international standard ISO 527–2 (49), for mechanical and thermal testing and Fourier-transform infrared spectroscopy as detailed in Theobald et al. (50). Additional paddles (rectangular plates with integrated arm) were used for surface microstructure analysis by scanning electron microscopy. Briefly, marginal effects of UV-aging were reported for PET and moderate changes for LLDPE, PA, and PLA. Artificially aged oxo-LLDPE paddles had increased crystallinity, intense surface cracking, and deterioration of their mechanical properties following 52 weeks of deployment in the pond water.

The structure was deployed during austral winter on 27 July 2020, and the final sample was collected on 28 July 2021. During sampling, plastic paddles were excised in triplicate (one per plastic per depth, i.e., 20, 40, and 60 cm from the water surface) at 2, 6, 26, and 52 weeks. Additionally, glass “control” samples were collected in triplicate, one per depth, at each sampling time. In total, 18 paddles (including the glass) were collected per sampling time (*n* = 72 in total), noting that the top (20 cm) glass sample was lost during final sampling (52 weeks); therefore, only two glass replicates were sampled at 52 weeks (*n* = 71 in total). An additional set of samples was collected from a duplicate set of deployed plastics for analysis of the chemical composition of the plastics and biofilms that developed on them, but with analyses only being completed on samples extracted after 26 and 52 weeks of pond water immersion. To collect paddles, sterile sample bags (Thermo Fisher Scientific, Waltham, MA, USA) were carefully placed over each paddle before being excised from the structure at the integrated arm using pipe cutters. In addition to glass controls, three 2 L pond water samples were collected at approximately 20–40 cm from the water’s surface near the structure. Water was collected in acid-washed and autoclaved 2 L Schott Duran glass bottles. Each bottle was rinsed with the maturation pond water three times before sample collection. All samples were placed on ice immediately upon collection and transported to the laboratory for processing. Samples for biofilm removal were processed upon receipt at the laboratory, while samples for chemical analysis were stored at −20°C until required for processing.

### Processing biofilm and pond water microbial communities for molecular analysis

Biofilms were extracted from the plastic paddles and glass samples as per Wallbank et al. (10). A sterile flat-edged razor blade was used to scrape off most of the biomass from both sides of the plastic and glass samples, placing the biomass material into separate sterile 50 mL tubes (Cat No. 227–261; Greiner Cellstar, Sigma-Aldrich, St. Louis, MO, USA). The paddles were then placed back into their sample bags along with 30 mL of ice-cold, sterile Tris EDTA buffer (TE; Tris 10 M EDTA 1 mM, pH 8.0) and sonicated for 2 min at 35 kHz to recover biomass fixed to the paddles (Bandelin Sonorex RK 100H; Sigma-Aldrich, St. Louis, MO, USA). The sonicated solution was added by pouring it into the recovered biomass and centrifuged at 4,500 × *g* for 10 min at 4°C. The supernatant was decanted, and the pelleted material was centrifuged again, removing any additional supernatant via pipette. To concentrate microbial biomass from the pond water, roughly 300 mL of each pond water sample was filtered through a different 0.2 µm filter membrane (Supor 200; Whatman, Maidstone, UK) using a vacuum pump. Membranes were then placed in individual, sterile 5 mL centrifuge tubes. All biofilm samples were stored at −80°C until further processing.

### Processing plastic and biofilm material for the analysis of trace elements

To determine the inorganic content of the plastics, TGA was carried out in triplicate per sample type, on a TGA1-0329 instrument (TA Instruments, USA). Samples of plastic (10–30 mg) were placed in 100 µL high-temperature platinum sample pans and heated by 10°C per minute, from ambient temperature to 800°C under a nitrogen atmosphere with a 10 mL/min flow rate. Samples were then held at 800°C for 10 minutes while exposed to a 10 mL/min air flow rate. The weights of plastics before and after heating were then compared.

Biofilms for chemical analysis were removed by freeze-drying the paddles and scraping off the biological growth using a sterile plastic paddle of the same plastic type as the sample. The freeze-dried biofilm and paddles were stored at −20°C until required. Biofilm material was manually homogenized before analysis. The approach of Masterton (51) was employed to quantify the trace elements of the plastic paddles and associated biofilm material. Plastic paddle (0.2 g) and biofilm (0.4 g) samples were digested with 5 mL of 69% ultrapure nitric acid in an UltraWAVE single reaction chamber microwave digestion system for analysis by Inductively Coupled Plasma-Mass Spectrometry (Agilent 8900 Triple Quadrupole ICP-MS). Rhodium was added online as the internal standard, with oxygen and helium used as the reaction and collision gases to remove interferences. Sample extracts were diluted with 0.05% tartaric acid solution for analysis and a second dilution with 2% HNO_3_/1% HCl for mercury analysis. Replicates of two certified reference materials, ERM-EC68 Low-density polyethylene and INCT-OBTL-5 Tobacco leaves, were analyzed alongside the samples; trace element recoveries were acceptable (recovery ranges of 72%–105% and 73%–112%, respectively). Plastic paddles of the same composition as the deployed plastics were similarly analyzed to assess seawater immersion’s impact on each plastic type’s chemical attributes.

### DNA extraction

DNA was extracted from a total of 83 samples [triplicate samples per time point (4), i.e., 12 biofilm samples per plastic type (five plastic types), 12 pond water samples, and 11 glass biofilm samples], alongside sampling blanks and DNA extraction kit control blanks. Microbial DNA was manually extracted from up to 250 mg of biomass using DNeasy PowerSoil Pro kits (Qiagen, Hilden, Germany), according to the manufacturer’s instructions, except for the mechanical lysis step, which was performed using a TissueLyser II (Qiagen, Hilden, Germany) for 2 min at 30 Hz. UltraPure DNase/RNase-Free Distilled Water (100 µL; Invitrogen, Thermo Fisher Scientific, Waltham, MA, USA) was used to resuspend any pelleted samples with <100 µg of biomass. The entire volume was used for the extraction. Filtered pond water samples were placed directly into bead tubes using sterile tweezers. Finally, DNA was eluted in 100 µL elution buffer as per the manufacturer’s instruction. DNA quality was assessed using an Implen Nanodrop photometer (Munich, Germany), and DNA concentrations were assessed using a Qubit double-stranded DNA High-Sensitivity Assay Kit (Thermo Fisher Scientific, Waltham, MA, USA). DNA samples were aliquoted and stored at −80°C until required.

### Shotgun metagenomic DNA sequencing

To determine the functional potential of microbial communities, metagenomic sequencing was undertaken on 83 samples. Thruplex DNA libraries were prepared by Otago Genomics (University of Otago, New Zealand) using NextSeq 2000 P3-300 reagent kits (Illumina Inc, Calif., USA) for 2 × 150 bp paired-end sequencing using an Illumina NextSeq 2000 Instrument.

### DNA sequence data processing and annotation

Raw metagenomic sequence reads were quality-filtered and trimmed using Trimmomatic (v 0.39-Java-1.8.0_144) (52). Sequence reads were discarded if there was a >2 bp mismatch, the paired read score was below 40, or the single read score was below 15. Universal Illumina adapters added by the sequencing provider were removed from sequence reads as per TruSeq3-PE-2.fa, and leading and tailing ends were removed if there was a quality score <3. Trimmed read quality was assessed using FastQC v0.11.7 (53). Trimmed reads (forward and reversed) were merged into a single interlaced file using the fq2fa tool from IDBA-UD [v 1.1.3; (54)]. Prodigal [v2.6.3; (55)] was used to predict open reading frame (ORFs) of trimmed and merged reads, and DIAMOND v2.0.15 (56) was used to align ORFs against a non-redundant orthologous protein reference database (NCBI-nr accessed 12.9.22) (57). MEGAN6 (58) was used to determine the functional content of ORFs against the SEED database (February 2022 version via MEGAN6) to predict general patterns in microbial function (59). The KEGG (Kyoto Encyclopedia of Genes and Genomes) database (May 2018 version) was used, with the DIAMOND default *e*-value of 0.001 taking the top hit above 50% identity match to investigate differences in the abundance of critical biological pathways (60).

### Data analysis

The overall experimental design consisted of 72 samples assigned to three factors: substrate (six substrates: LLDPE, PA, PET, PLA, oxo-LLDPE, and glass), depth (three levels: top, 20 cm from the surface of the pond water; middle, 40 cm from the surface of the pond water; and bottom, 60 cm from the surface of the pond water), and sampling time (four levels: 2, 6, 26, and 52 weeks after deployment installation at the study site). Pond water community data were included in some analyses (three samples per sample depth and time; *n* = 12 additional samples). All quantitative and statistical analyses and data visualization were performed using R (version 4.2.2) (61). Taxonomic abundance tables were merged and normalized using cumulative sum scaling (CSS) before statistical analyses, as suggested by Paulson, Stine (62).

#### Taxonomic assignment

Taxonomic information was obtained using the default databases and parameters of Metaxa2 (63). Partial rRNA sequences from archaeal, bacterial, and eukaryotic origins were extracted from trimmed and merged reads. Sequences from taxa that were not identified as belonging to any of these three domains were removed, along with all singletons. We used Metaxa2 to classify reads based on the SILVA database (http://www.arb-silva.de). Pairwise permutational multivariate analysis of variance [PERMANOVA; (64)] with 999 permutations was carried out on Bray-Curtis dissimilarity matrices using the vegan “adonis” function (65) to statistically evaluate whether sampling time, depth, and substrate impacted sample community composition. Community clustering was visualized on a non-metric multidimensional scaling plot (NMDS) using the “vegdist” function and Bray-Curtis dissimilarity metric. Because no statistical differences were observed among substrate types, substrate-specific data were pooled when showing taxonomic relative abundances as bar plots. Taxon relative abundance was used when investigating the top five most abundant bacterial genera in different sample groups.

#### Functional assignment

Broad-scale functional SEED and KEGG data were filtered and normalized as per the taxonomic data. One PA sample was removed due to low assignment counts in both data sets. Bray-Curtis dissimilarity matrices of the data and PERMANOVAs with 999 permutations were used to compute SEED and KEGG functional potential. Gene fragment abundances assigned with second-level KEGG assignments were adjusted to maximum-minimum abundance instead of relative abundance to display which functions are most abundant across all substrates and which function is most abundant per substrate. Using permutational multilevel pattern statistical analysis (*P* < 0.05), indicator values for sequence reads assigned to second-level KEGG assignments were compared to identify functions representative of a sample community. Indicator functions were assigned to either substrate or depth, with significance noted by the default parameters of the permutation test from the indicspecies package (66). Venn diagrams were built using the VennDiagram package, showing the number of functional indicator overlaps at second-level KEGG assignment.

#### Plastic degradation and antimicrobial resistance

To explore the presence of specific functional traits, trimmed and filtered amino acid sequences were compared to those banked in a database of putative plastic-degrading taxa and associated enzymes (PlasticDB.org) (67) and to a comprehensive database of genes relating to microbial antibiotic resistance (68). Functional data were compared with the plastic database using BLASTP local alignment to retrieve the total counts of related sequences. Antimicrobial resistance data were CSS normalized and visualized on an NMDS plot, based on Bray-Curtis dissimilarity. The “envfit” function was used to fit the most abundant antibiotic genes (>1% depth relative abundance) as factors onto the ordination. As seen for the taxonomic data, PERMANOVA revealed no significant difference between substrates. Finally, antimicrobial resistance gene sequences were aligned using MAFFT v7.505 (69) and assembled into a phylogenetic tree using FastTree v2.1.10 (70) based on the Le and Gascuel model of amino acid alignment (71). The resulting antimicrobial resistance gene tree was visualized using the iTOL v6 online tool (72).

## RESULTS

A significant difference in average water temperature was detected among sampling times, ranging from an average of over 8.7°C in July to *c*. 18.9°C in January (single factor ANOVA [analysis of variance], *F* = 2484.7, *P* = <0.01), but no significant difference was detected among the three depths (single factor ANOVA, *F* = 0.005, *P* = 0.995). Light intensity ranged between 318 and 4,590 lux (Table S1), showing a contrasting relationship with temperature, with a significant difference related to depth (single factor ANOVA, *F* = 77.48, *P =* <0.01), but no significant difference among sampling times (single factor ANOVA, *F* = 0.05, *P* = 0.993).

### Plastic and biofilm inorganic composition

#### Inorganic and plastic additive content quantified before environmental deployment

We detected no significant concentration of inorganic matter within the different plastic formulations used in this study. The inorganic content of the plastic paddles before being deployed in the environment was below the detection limits of the TGA for PLA, PA, and PET, which is ±0.1% of the original mass of the samples (Table S3). The final residual mass was slightly higher for oxo-LLDPE and LLDPE, at 0.1%. The total additive content for each of the plastics was <1% of their final absolute weight (Table S2).

#### Changes in the trace element content of the plastic paddles

Only five elements significantly increased in concentration in some plastics over time: aluminum (Al) in PLA (single factor ANOVA, *P* = 0.07, *F* = 5.14); chromium (Cr) in LLDPE, oxo-LLDPE (*P* = <0.01, *F* = 5.14), and PLA (*P* = 0.055, *F* = 5.14); iron (Fe) in LLDPE (*P* = 0.07, *F* = 5.14) and oxo-LLDPE (*P* = <0.01, *F* = 5.14); manganese (Mn) in oxo-LLDPE (*P* = 0.07, *F* = 5.14), PET, and PLA (both *P* = 0.02, *F* = 5.14); and phosphorus (P) in oxo-LLDPE, PET (both *P* = <0.01, *F* = 5.14), and PLA (*P* = 0.02, *F* = 5.14; Table 1). No element significantly increased in concentration over time in PA. While not statistically significant, LLDPE and oxo-LLDPE also appeared to increase in P, Mn, and lead (Pb) concentrations (Table 1), with oxo-LLDPE increasing in nickel (Ni) concentration (Table S4). LLDPE formations also had surprisingly high levels of zinc (Zn), previously not disclosed by the manufacturers (Table S4). Additionally, PET had increases in Mn and copper (Cu), PLA had increases in Ni, and PA had increases in Cr, Mn, Fe, and Pb concentration (Table 1). The remaining tested elements fluctuated around the same values across the experimental period or had a maximum value at 26 weeks (Table S4). No elements were found to decrease significantly in concentration over time.

**TABLE 1:** Average trace element concentration (mg/kg) within the plastic paddles^*a*^

	Detection limits (mg/kg)	LLDPE plastics		Oxo-LLDPE plastics		PET plastics		PLA plastics		PA plastics	
Time		t0	t52	t0	t52	t0	t52	t0	t52	t0	t52
Al	7.5	95.8	95.7	87.0	76.0	12.4	15.9	15.2	8.3	43.2	27.0
P	0.8	87.4	141.0	46.4	118.8	16.4	38.9	0.2	14.8	1.1	31.0
Cr	0.1	ND^*b*^	0.2	ND	0.3	0.2	0.2	ND	0.1	0.1	0.3
Mn	0.8	0.1	7.7	91.0	123.0	ND	5.5	ND	5.9	0.1	8.4
Fe	7.5	9.6	25.8	3.9	35.0	2.2	12.2	5.1	13.5	9.0	24.8
**Pb**	0.8	ND	0.1	ND	0.1	ND	ND	0.1	ND	ND	ND

^*a*^ Data are shaded in dark gray when there were significant changes over time as per single factor ANOVA (P < 0.05). Data are shaded in light gray when there is a non-significant change.

^*b*^ ND, not detectable using current methods.

#### Change in the trace element content of plastic-associated biofilms

We determined whether the trace element content of plastics was enriched in associated biofilms. The trace element content of plastic-associated biofilms changed in composition over time, irrespective of the changes in trace element content of plastic paddles. Of the trace elements that increased significantly in concentration in the plastic paddles, Mn, an additive in the oxo-LLDPE (Table 2), more than doubled consistently across all plastic-associated biofilms, whereas Cr decreased in concentration across all biofilms (Table 2). Additionally, Fe and P concentrations consistently increased between 20% and 150% across all plastic-associated biofilms, whereas Al increased in concentration in PA and oxo-LLDPE biofilms by 83% and 46%, respectively (Table 2). In addition to significant changes in the inorganic content of plastic paddles over time, arsenic (As), strontium (Sr), and barium (Ba) concentrations all increased in the plastic-associated biofilms, with the maximum increases occurring in PA-biofilms for all three elements (Table S5). Ni was the only element to decrease in concentration consistently across all biofilms, with molybdenum (Mo) concentrations decreasing in all biofilms aside from oxo-LLDPE’s biofilm (Table S5). Cu, cerium (Ce), mercury (Hg), and Pb increased in concentration within select plastic biofilms: Cu and Hg increased by >25% in PET and oxo-LLDPE biofilms, Ce increased by >50% in LLDPE, PA, and oxo-LLDPE biofilms, and Pb increased by >70% in PA, PET, and oxo-LLDPE biofilms. Both PA and oxo-LLDPE biofilms had the greatest overall increase in trace element content. In contrast, PLA had the least overall increase in trace element content. All other elements either showed little to no change in concentration or were not detected in the biofilms (Table S5).

**TABLE 2:** Average trace element concentration (mg/kg) within the biofilms^*a*^

	Detection limits (mg/kg)	LLDPE biofilms		Oxo-LLDPE biofilms		PET biofilms		PLA biofilms		PA biofilms	
Time		t26	t52	t26	t52	T26	T52	t26	t52	t26	t52
Al	7.5	7955.3	9525.5	6697.3	9790.0	8706.4	9837.9	10696.1	10369.3	6143.4	11297.5
P	0.8	9224.1	12264.3	6464.6	11176.4	6463.0	11619.6	6451.0	9434.3	7830.1	10473.5
Cr	0.1	155.1	120.3	152.3	152.6	251.3	142.4	229.4	112.5	181.9	137.0
Mn	0.8	1593.2	3747.7	845.3	3178.8	542.8	3540.6	745.2	3068.3	506.9	3129.8
Fe	7.5	8341.7	16164.2	7223.0	16370.8	10136.2	15917.5	10939.9	15946.5	6722.4	16914.9
Pb	0.8	25.2	29.9	16.3	31.0	18.2	31.4	22.6	26.6	15.2	31.3

^*a*^ Biofilm data were not statistically verified due to lack of replicates. Data are shaded in dark gray to indicate changes in metal composition.

### Putative taxonomic diversity across metagenomic reads

**Fig 2:**
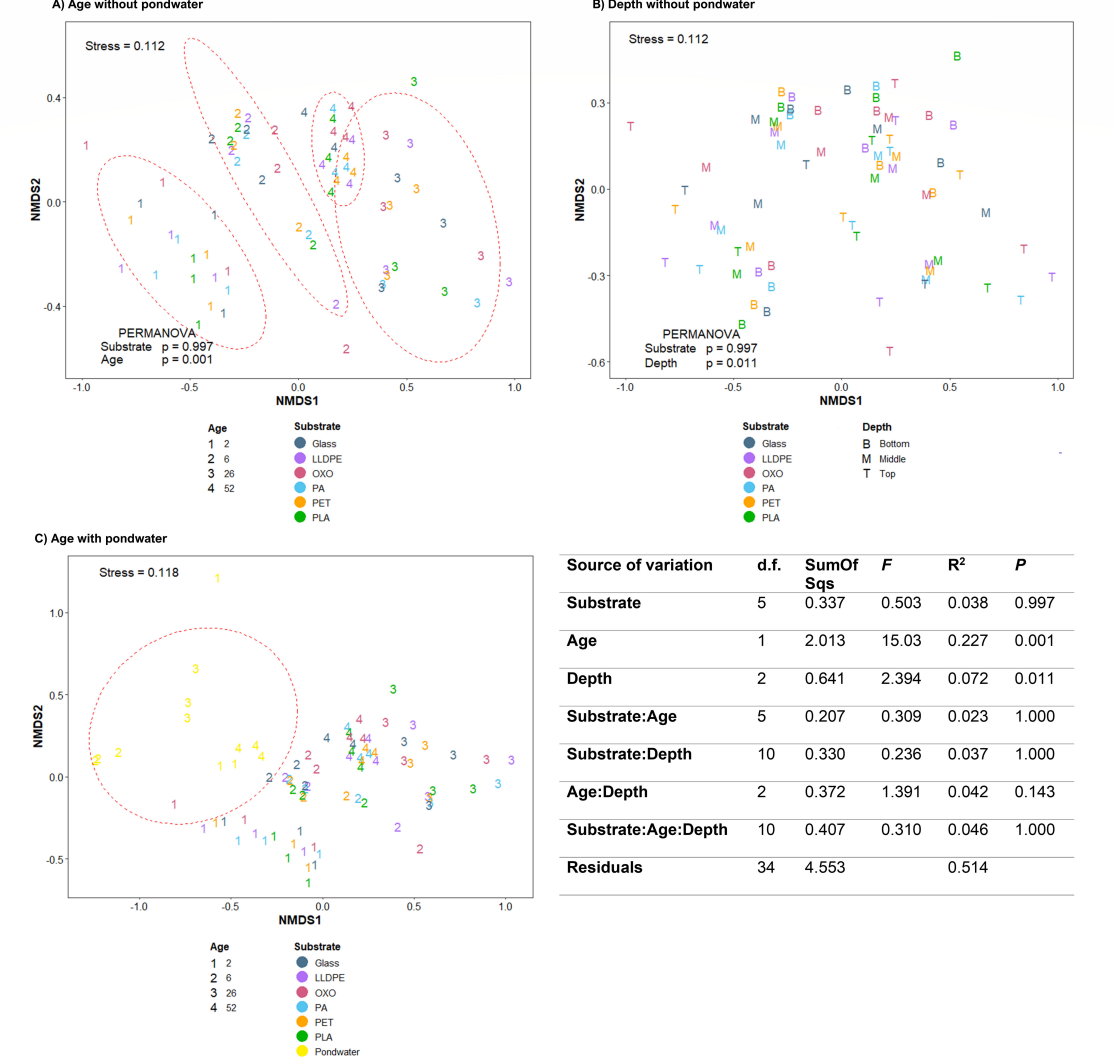
Variation in microbial community composition associated with different plastics after incubation at different depths within a wastewater treatment pond, based on analysis of unassembled shotgun small subunit and large subunit rRNA gene sequences. (**A–C**) (NMDS ordinations of data using the Bray-Curtis similarity measure, grouped by (**A**) biofilm age (in weeks), (**B**) depth below the water’s surface, at 20 cm intervals, and (**C**) by sample age, but with the inclusion of data from planktonic pond water communities. Substrates deployed consisted of a glass control and five plastic types: LLDPE, oxo-LLDPE, PA, PET, and PLA. Ellipses assume a multivariate *t*-distribution with an alpha of zero. Results of the PERMANOVA for the input data used in plots A and B, run using the adonis package in R, showing the partitioning of multivariate variation and tests for the factors of substrate, age, depth, and their interactions.

Biofilm community composition differed significantly among sample ages and depths (PERMANOVA, *P* = 0.001 and *P* = 0.011, respectively) but with no significant variation among substrate types (*P* = 0.997; Fig. 2). Pond water community data were the only “substrate” data to cluster independently of all other substrates (Fig. 2C). They were subsequently removed from analyses when determining the effects of age, depth, and substrate on community composition. Top-tier substrates (20 cm from the pond water surface) had the greatest variation in community composition (homogeneity of multivariate dispersion values relating to average Bray-Curtis distances within data collected from 20, 40, and 60 cm depth were 0.3607, 0.3243, and 0.3232, respectively), noting that by 52 weeks, data from the top tier clustered closely with data from the middle and bottom tiers (Fig. 2A and B). At all other times, there was minimal overlap between communities sampled at different depths. As signified by the greater *R*^2^ value, the age of the biofilm had the greatest effect on community composition (PERMANOVA, *P* = 0.001); there was minimal overlap among data from the different sampling times (Fig. 2A).

**Fig 3:**
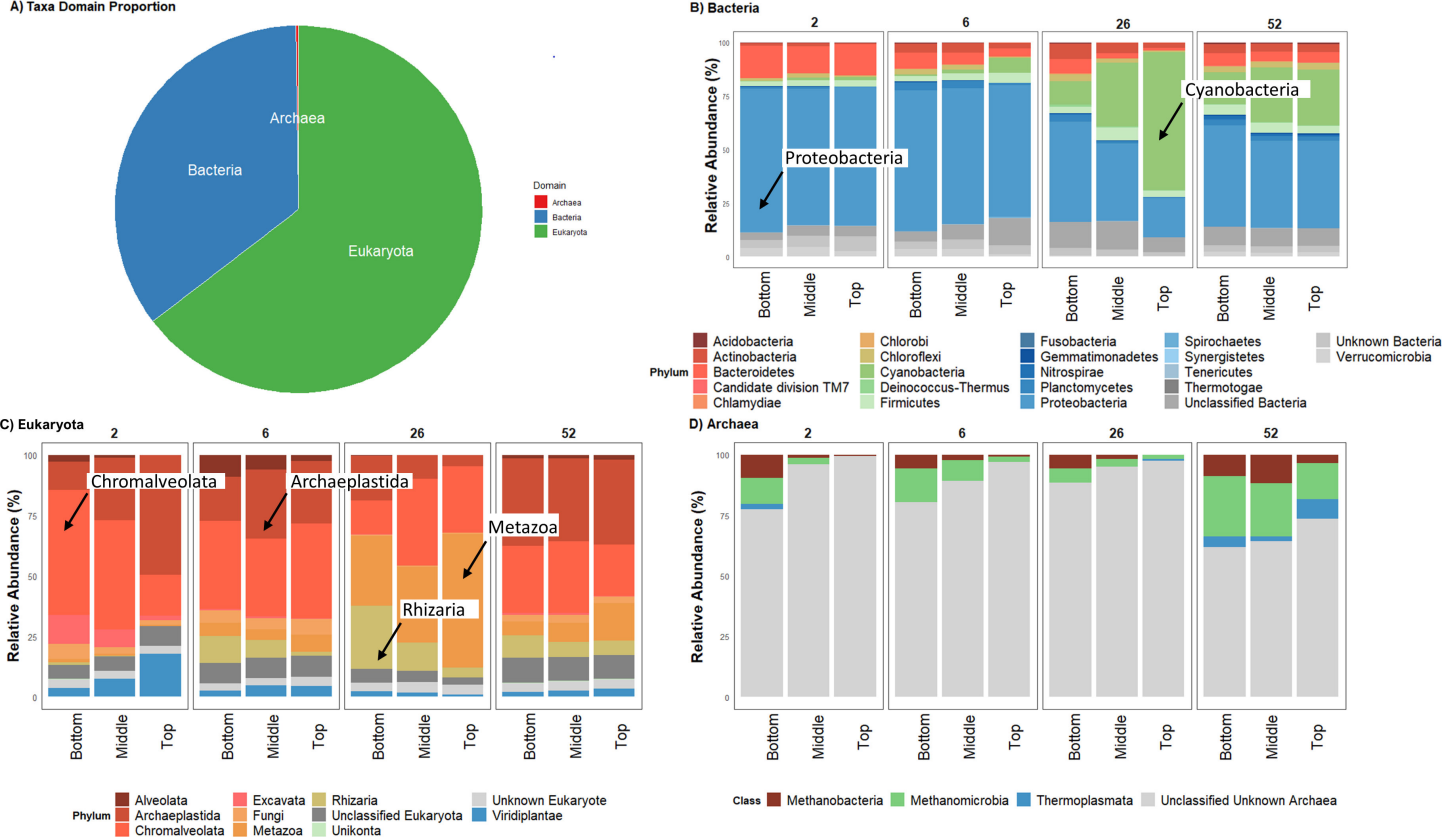
Relative abundance and proportion of SSU and LSU rRNA fragments recovered and successfully assigned to the three key domains of life using the default database of Metaxa2 (63). (**A**) Bacteria (prokaryote; 35.3%), eukaryotes (64.5%), and Archaea (0.2%). (**B–D**) The cumulative sum scale relative abundance for phylum (**B and C**) and class (**D**) taxonomic assignments for Bacteria, eukaryotes, and Archaea, respectively. Plastic-associated biofilms were sampled from waste-water treatment plant maturation ponds. Data obtained from the different plastic substrates were averaged since we confirmed no significant difference in community composition. The sample data presented here came from all substrates, including LLDPE, oxo-LLDPE, PA, PET, PLA, and glass as a control; pond water sample data were not included in these figures.

Total short subunit (SSU) and long subunit (LSU) rRNA gene fragments (*n* = 956,125) recovered using Metaxa2 (63) resolved reads to bacteria (35.3%), eukaryotes (64.5%), and archaea (0.2%) after all unidentified and chloroplast data were removed (Fig. 3A). *Proteobacteria* was noted as being the most dominant bacterial phylum. This was consistent across all times, substrates, and depths, except at the top tier in week 26, where *Cyanobacteria* were noted as being dominant; they remained abundant at 52 weeks (Fig. 3B). Eukaryotic community composition was less consistent across depth and time but was still dominated by a few common phyla/superclades (Fig. 3C). *Archaeplastida* and *Chromalveolata* dominated sequence reads at all sampling times, except for 26 weeks, where unclassified *Metazoa* and *Rhizaria* dominated. Additionally, a substantial proportion of unclassified eukaryotes were present at all sampling times. Interestingly, Fungi represented only a small proportion of the relative abundance of eukaryotic reads, around 5% of the total eukaryote DNA sequence data recovered. Finally, Archaea were the most poorly identified domain (Fig. 3D). Most of the reads characterized as Archaea had no phylum-level classification, while those identified exclusively belonged to the kingdom of *Euryarchaeota*. All classified Archaea reads remained at similar proportions for 2, 6, and 26 weeks, but at 52 weeks, *Methanomicrobia* became proportionally more abundant.

#### Evaluating Metaxa2 taxonomic assignment

To a lesser degree than Eukaryota and Archaea, a large proportion of bacterial rRNA gene fragments were unclassified at the genus level (*n* = 372, 15.14%), with a high proportion remaining unclassified at the phyla level. Additionally, there was little impact of substrate type on the composition of the top five genera (Fig. S1). A pairwise PERMANOVA revealed that communities on each substrate only differed significantly from pond water data (PERMANOVA, *P* = 0.004, *F* > 5.5). Sample depth and age were associated with differences in the composition of phyla and the top five genera (PERMANOVA for genera *P* = 0.042 and *P* = 0.001, respectively). Of the successfully characterized reads, *Flavobacterium* was one of the dominant genera at 2 weeks, with *Cyanobacteria* becoming more prevalent at 26 weeks (Fig. S1). *Aeromonas* were prominent in the top tier (i.e., shallowest depth) at 6 weeks for all samples except for PET-associated communities (Fig. S1). Conversely, pond water was dominated by different bacterial taxa than the biofilm-associated communities. *Acinetobacter*, *Aeromonas*, and *Flavobacterium* dominated the top tier at 2, 6, and 26 weeks, and 52 weeks samples were dominated by *Polynucleobacter*, which dominated the pond water samples at all times (Fig. S1).

Taxonomic assignments of unassembled metagenomic reads demonstrated a significant (*t* test for coefficients, *P* < 0.01) correlation, at the phylum level, with 16S rRNA amplicon data sourced from a similar amplicon-based study [unpublished data; SILVA version 138.1 (73); Fig. S2]. However, a similar regression analysis revealed a non-significant correlation at the genus level (*P* = 0.574, F = 0.3159, R^2^ <0.01, 1,112 df). Any higher taxonomic resolution resulted in more taxa not being shared among the data sets, likely due to different taxonomic synonyms used in each and the nature of unassembled metagenomic reads compared to targeted 16S rRNA gene region amplification, where analysis of different sequence regions can lead to differences in classification.

### Broad-scale functional assignments

**Fig 4:**
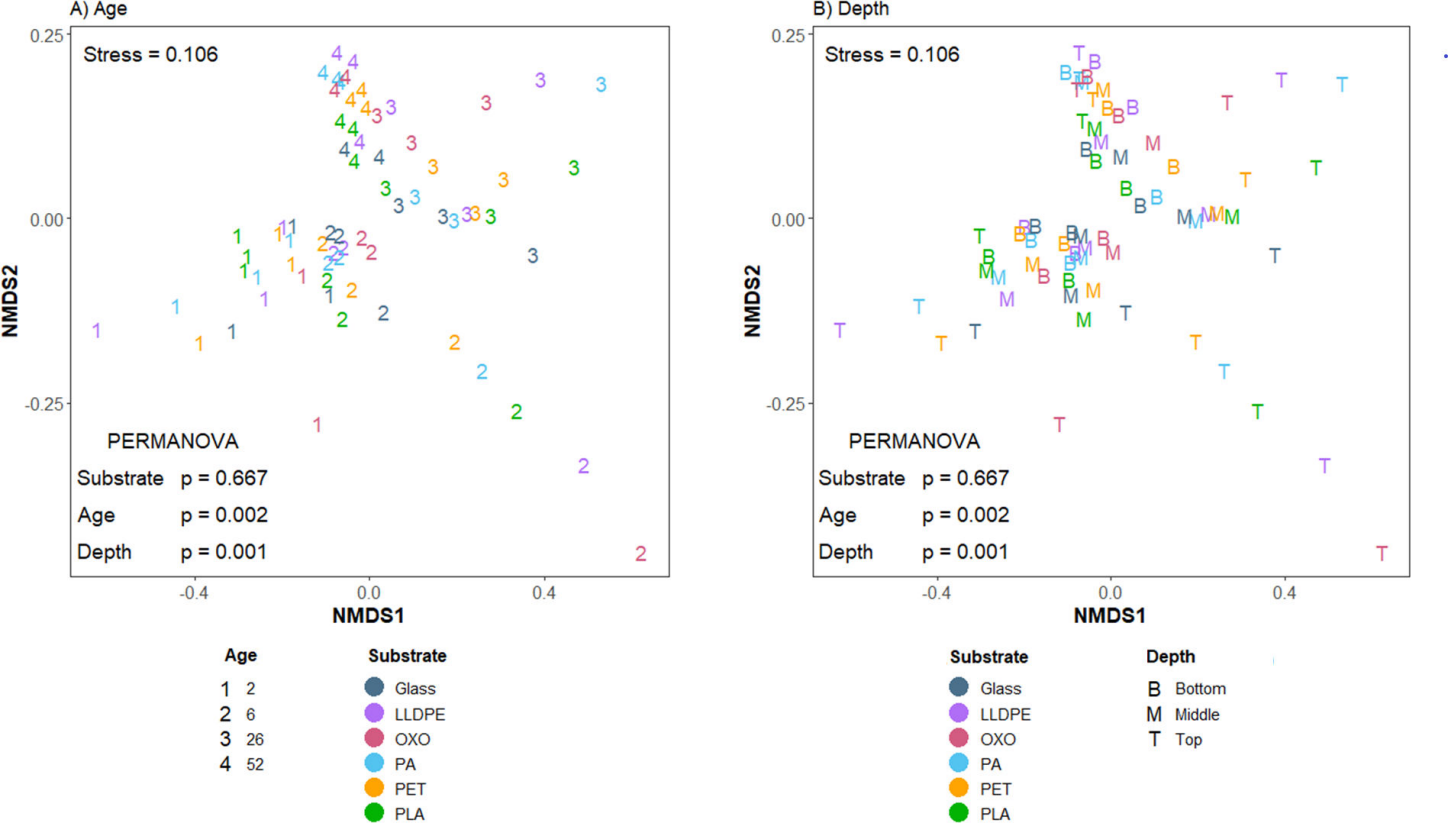
Underlying differences in SEED classification of plastic and glass-associated biofilm communities. Plots are NMDS ordinations of Bray-Curtis similarities of CSS normalized data, labeled by (**A**) sample age or (**B**) sample depth; LLDPE, oxo-LLDPE, PA, PET, and PLA.

The broad-scale functional potential of plastic-associated biofilms differed significantly among sample ages and depths (PERMANOVA, *P* = 0.002 and *P* = 0.001, respectively) but not with substrate type (PERMANOVA *P* = 0.667, excluding pond water data; Fig. 4). NMDS plots based on Bray-Curtis dissimilarities of SEED functional assignments revealed similar relationships in the functional data compared to the taxonomic data. Based on the homogeneity of multivariate dispersion analysis, biofilms closest to the pond water surface are the most diverse in terms of SEED functional assignments (distance from median 0.292, PERMANOVA *P* = 0.001). In contrast, middle- and bottom-tier biofilms remain more similar to each other (distance from median 0.073 and 0.220, respectively, *P* = 0.001; Fig. 4B). Similarly, with Metaxa2 assignments, as the biofilms age, i.e., at the last sampling time (52 weeks), depth stops being such a strong driver of the observed differences in diversity, with points representing data from all three depths clustering more closely (median Bray-Curtis distance at 2 weeks = 0.202, 6 weeks = 0.219, 26 weeks = 2.080, 52 weeks = 0.148, and PERMANOVA *P* = 0.002).

#### Broad-scale functions associated with inorganic element concentrations

**Fig 5:**
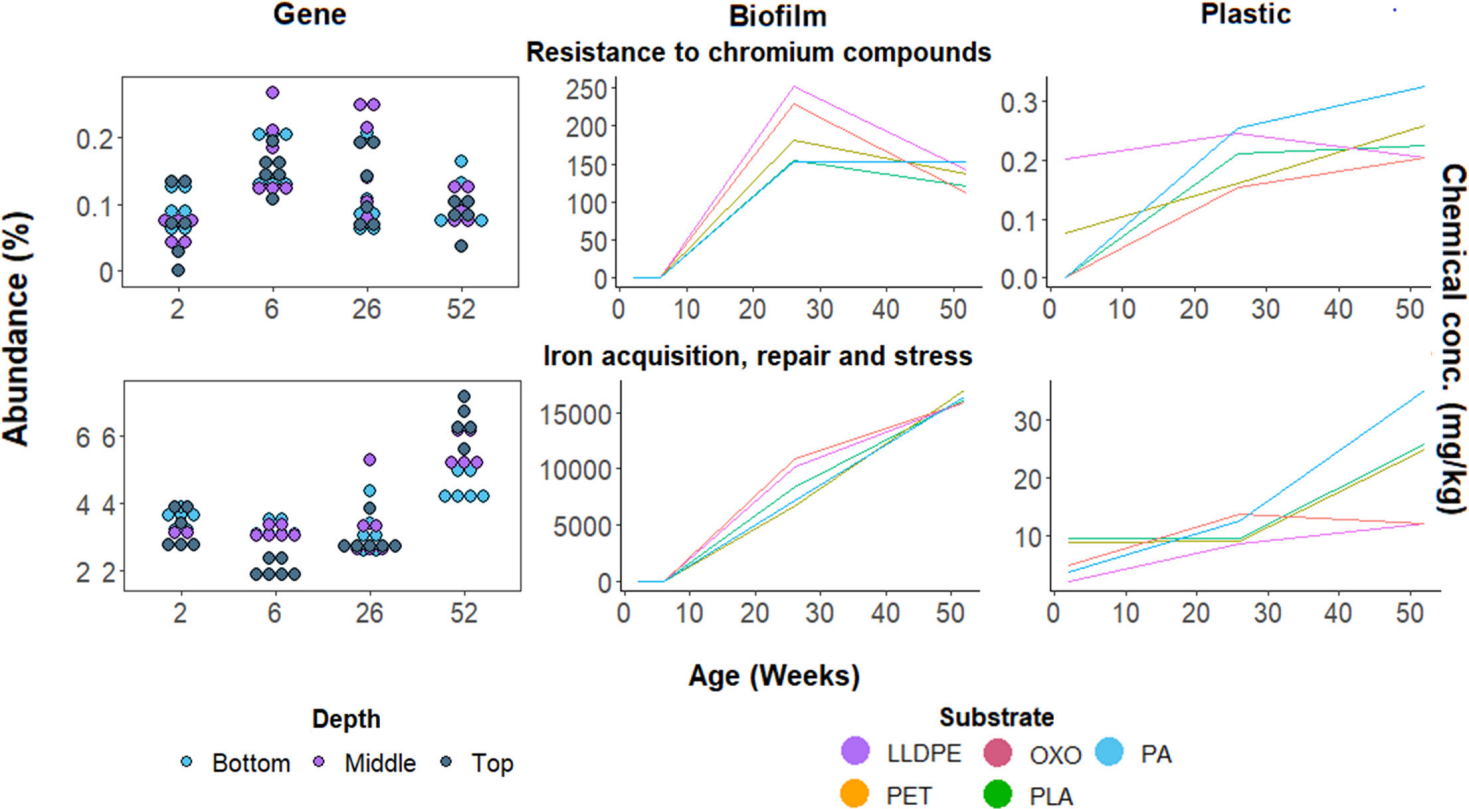
Relative abundance of genes associated with chromium (Cr, upper plots) and iron (Fe, lower plots), and metal concentrations within the plastic paddles and plastic-associated biofilms. Plots on the left show changes in gene abundance through time and by the depth of the substrate [top (20 cm from the surface), middle (40 cm from the surface), and bottom (60 cm from the surface)]. Each line in the middle and right panels represents metal concentrations in the biofilm (middle) or plastic substrate (rightmost) from one of the five plastics; data collected from different water depths were averaged. Additional metal abundance and associated gene data are presented in Fig. S3.

We considered six functions in the biofilms encoding for metal homeostasis, metal resistance, DNA and iron center protein repair, and stress response, particularly relevant considering the elements we identified in the plastic paddles and their associated biofilms (Fig. S3). These included functions related to Cr resistance, As resistance, Ni requisition and homeostasis, Zn requisition and homeostasis, Cu homeostasis, and Fe repair and stress. Concerning the two elements that significantly increased in concentration in the plastic paddles over time (Fe and Cr), we found Fe repair and stress genes increased in relative abundance with biofilm age (Fig. 5). Genes associated with Cr resistance increased in abundance at 6 and 26 weeks, before decreasing again around 52 weeks. Changes in the relative abundance of Cr resistance genes appeared to follow the same temporal trajectories as changes in Cr concentration in plastic-associated biofilms. Additionally, as As increased in concentration in plastic-associated biofilms (from below detection limits, on average, to 6.14 mg/kg), the relative abundance of As resistance genes also increased (<0.2% to ~0.4%; Fig. S3). The abundance of some metal-related genes, such as Cu-related genes, varied more at earlier sampling times (<0.2% to >0.6% at 2 weeks; >0.2% to <0.5% at 52 weeks). Conversely, the relative abundances of other metal-related genes, such as Ni requisition and homeostasis-related genes, increased in variance with time (~0.1% to 0.3% at 2 weeks; ~0.2% to 0.5% at 52 weeks), with biofilms at the bottom depth being most abundant in Ni-associated genes (~0.5%; Fig. S3).

### Relationship between KEGG assigned functional potential and substrate type, age, and depth

Metabolism and environmental signal processing were among the most dominant functional assignments across all substrates, depths, and ages (Fig. S4), most noticeably carbohydrate metabolism, energy metabolism, and amino acid metabolism. Genes encoding for cell growth and death, cell-cell signaling molecules and interaction, and membrane transport were also highly abundant. The age of the biofilm had the greatest impact on their relative abundance. Functional assignments associated with cellular processes, environmental information processing, genetic information processing, and metabolism were most abundant after 2 weeks of immersion. However, samples collected after 26 weeks were noted as having higher abundances of functions associated with cell communication, cell growth and death, and cell motility, particularly in comparison with 6 and 52 weeks. Twenty-six-week samples were also noted as having a higher abundance of sequences encoding for the production of drugs, which include antibiotics, nervous system agents (e.g., anxiolytics—used as minor tranquilizers), skeleton-based (e.g., naphthalenes), structure-based (e.g., prostaglandins), and target-based (various receptor based inhibitors) classification pathways. However, pond water appeared to have a higher number of more abundant functional assignments than other substrates at 6, 26, and 52 weeks.

**Fig 6:**
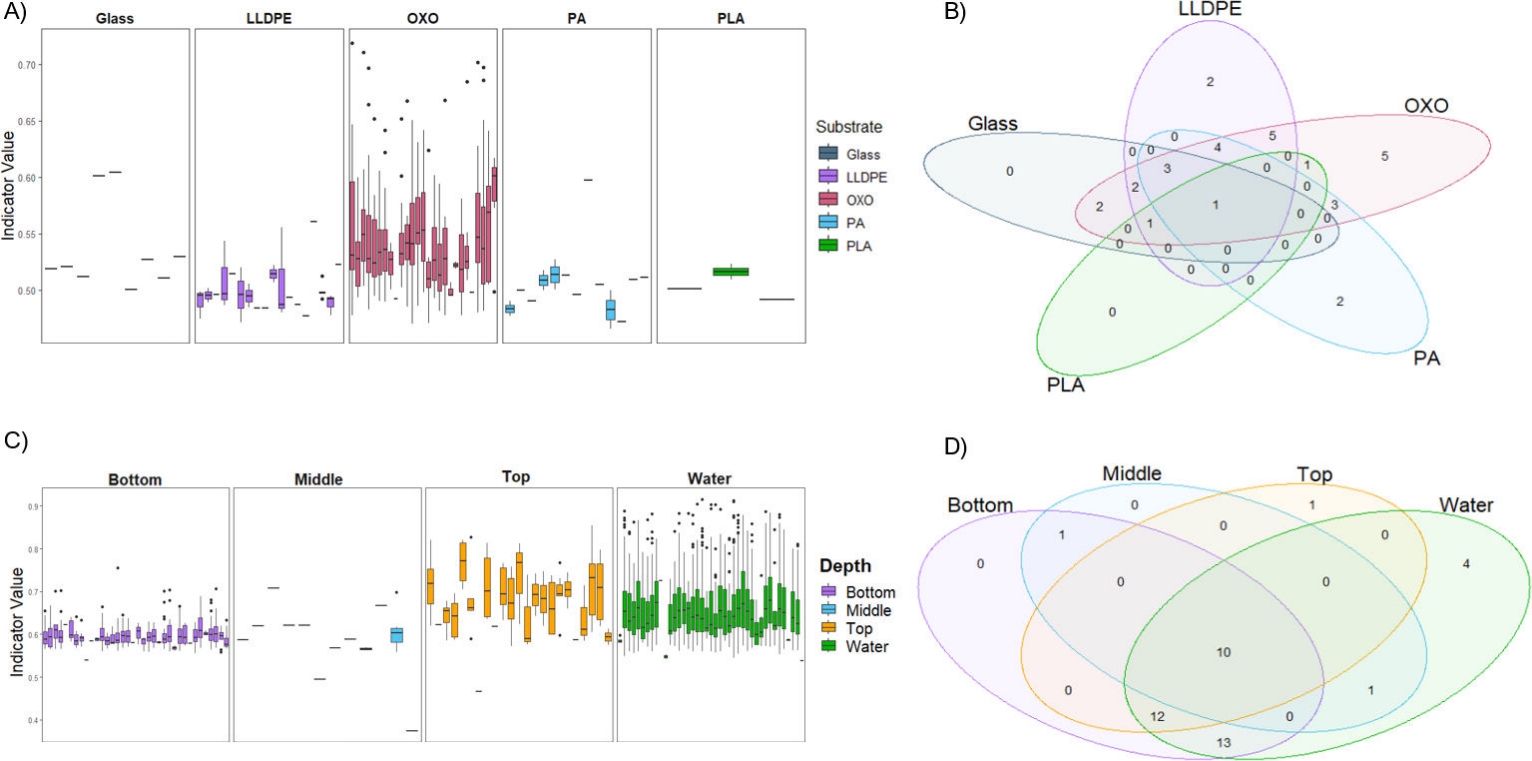
Distribution of multilevel pattern analysis to reveal functional indicator coding sequences across plastic-associated biofilms associated with different (A and B) substrates and (C and D) submersion depths. The classification was denoted by second-level KEGG functions of unassembled shotgun metagenomic sequences. Box and whisker plots display the quartile distribution of the indicator value assigned to each significant second-level KEGG function (*P* < 0.05). Whiskers indicate the spread of the upper and lower quartile, and dots denote the extremes of individual functions. X-axis labels were removed due to the high number of KEGG functions. Venn diagrams denote the number of unique or similar second-level KEGG functions associated with either substrate (B) or depth (D). Substrates include glass, LLDPE, PA, PET, PLA, and oxo-LLDPE. Three depths were sampled at 20 cm intervals from the surface of the final oxidation pond of a WWTP, with “top” being the closest to the surface and “bottom” the deepest. “Water” = pond water control. Predicted coding sequences were considered significant with an alpha value of 0.05, a minimum predicted positive value of 0.4, and a minimum sensitivity of 0.5. Indicator values were identified using the indicspecies packing in R (https://emf-creaf.github.io/indicspecies/) (74).

Several KEGG assignments were identified as indicators of sample depth and substrate type, except for PET. Indicators are functions that have a statistically significant (multivariate permutation) association with an experimental variable. When pond water data were removed, oxo-LLDPE biofilms contained the most KEGG-assigned groups, with 27 second-level classifications identified as indicators (Fig. 6A and B). PLA had the fewest classifications, with three comprising “energy metabolism,” “immune system,” and “metabolism of cofactors and vitamins” (Fig. 6A and B). LLDPE, oxo-LLDPE, and PA shared four second-level functional assignments not shared with glass or PLA (Fig. 6A and B). This is largely due to the high number of functional assignments found on oxo-LLDPE over any other substrate type and the comparatively low number of assignments found to indicate growth on glass and PLA. Glass and PLA had no exclusive second-level classifications. In contrast, the other plastic substrates had at least some functional assignments exclusively found (Fig. 6A and B).

When comparing the depth of biofilms with pond water data, pond water data contained the most indicator values for second-level KEGG assignments (Fig. 6C and D). In contrast, biofilm communities submerged 40 cm from the pond water surface (middle depths) contained the lowest number of assignments (Fig. 6C and D). Only two second-level KEGG assignments were found on the substrates and not detected in pond water. These were sequences encoding pathways involved in producing antibiotics, found exclusively on the top-situated substrates, and sequences encoding pathways involved in producing nervous system agents, indicative of multicellular organisms, or their DNA, found in or on the biofilms during sampling.

### Presence of previously reported putative plastic degradation genes

**Fig 7:**
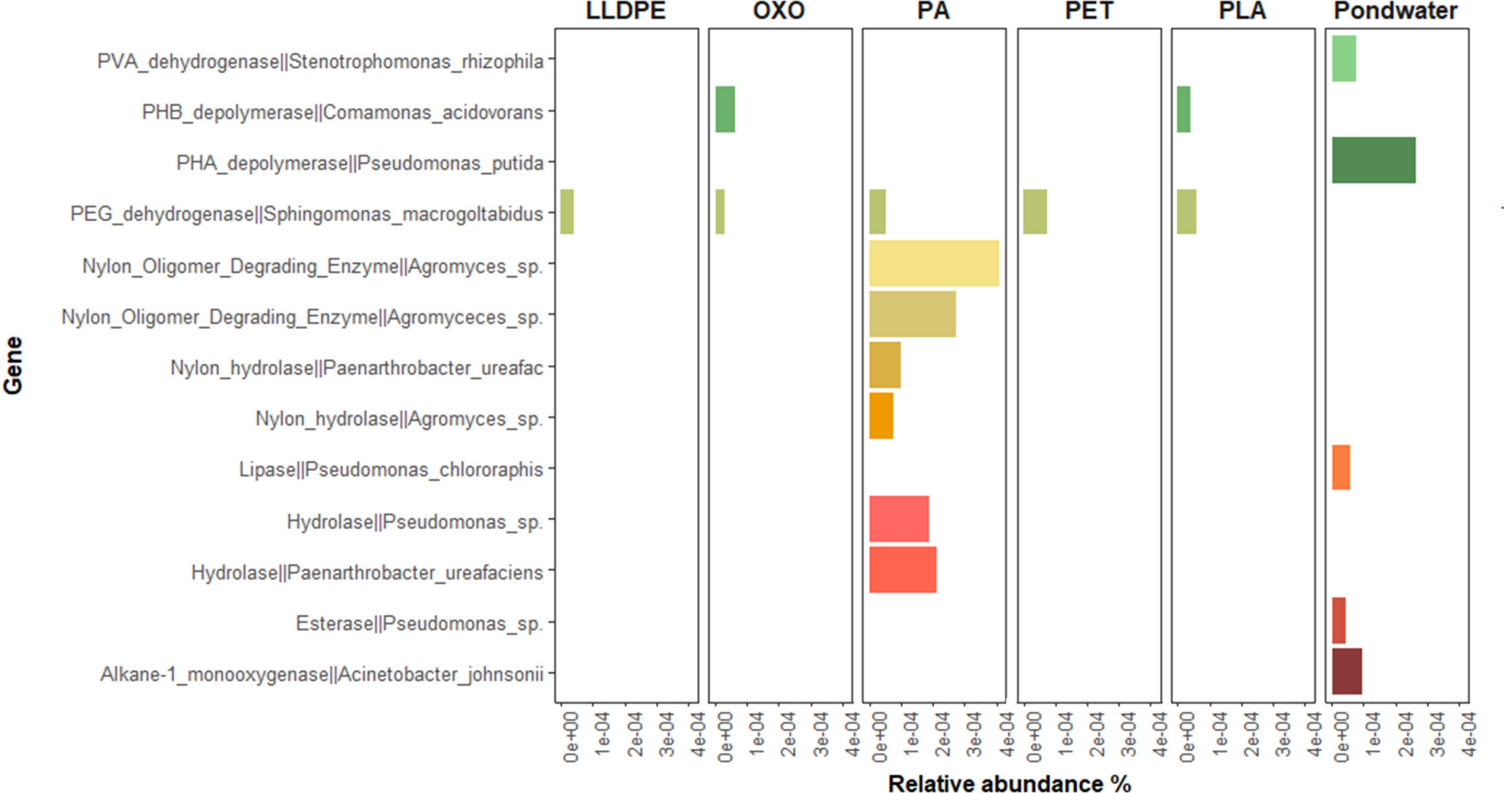
Relative abundance of genes previously reported as being involved in plastic biodegradation, based on a 95% blastp identity match, and with at least five or more reads identifying to a designated protein. Protein sequences encoding plastic biodegradation were sourced from PlasticDB.org (67). Total counts are the sum across all substrate biofilm replicates.

Overall, there was a low number of reads that were successfully assigned to putative plastic biodegradation proteins (*n* = 476; Fig. 7). The collection of pond water reads had several fragments assigned to various proteins reported as being linked to plastic degradation, such as polyhydroxyalkanoate (PHA) depolymerase, polyvinyl alcohol (PVA) dehydrogenases, lipases, esterases, and alkane-1-monooxygenase, all with a 95% identity match. Pond water data contained a higher richness and abundance of putative plastic-degrading genes than all biofilms, except for PA. PA biofilms generated the most sequences associated with plastic degradation, including those encoding nylon oligomer-degrading enzymes and hydrolases. Communities on the remaining substrates, in addition to PA, contained genes encoding the polyethylene glycol dehydrogenase protein found in *Sphingomonas macrogoltabidus*, while PLA- and oxo-LLDPE-associated communities encoded for a PHB depolymerase found in *Comamonas acidovorans*. No putative plastic-biodegrading protein-encoding genes were found on glass samples.

### Presence of antimicrobial resistance gene families

**Fig 8:**
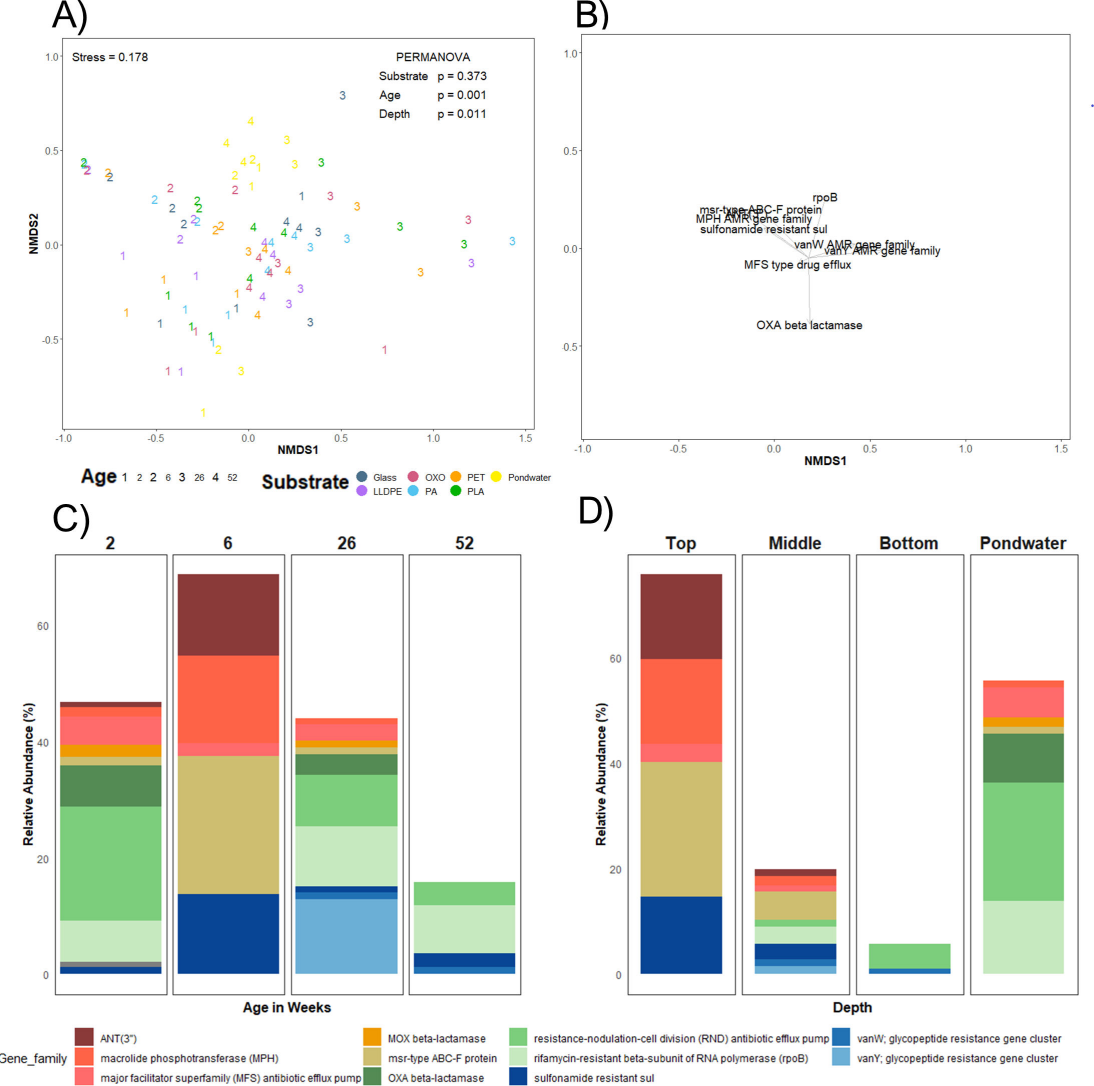
Variation in the composition of AMR gene families among plastic-associated biofilm communities. (**A**) NMDS ordinations of Bray-Curtis similarities of CSS normalized data, organized by the age of the biofilm by weeks, and substrate type (PERMANOVA: age *P* < 0.01 and substrate *P* = 0.37); LLDPE, oxo-LLDPE, PA, PET, and PLA, with (**B**) gene families, plotted as vectors. Changes in the relative abundances of AMR gene families above 1% relative abundance, according to (**C**) biofilm age (2, 6, 26, and 52 weeks) and (**D**) depth of the biofilm from the maturation pond water surface (top = 20 cm from the surface, middle = 40 cm, bottom = 60, and ambient maturation pond water).

Mobile genetic elements, such as those involved in conjugation, can be linked with AMR traits. These traits were not differentially abundant when comparing communities on the solid substrates with those from the pond water (Fig. S5). However, pond water had an increased relative abundance of conjugative transfer genes at 52 weeks, which was not observed for the biofilm communities (Fig. S5). AMR genes differed in both abundance and richness between biofilms and pond water controls. The pond water has a greater richness and abundance of AMR genes than middle and low-tier biofilms (Fig. 8). Top-tier biofilms were differently abundant in AMR gene families compared to pond water controls. Community AMR gene family composition differed significantly based on the age and depth of the biofilms (PERMANOVA *P* = 0.001 and *P* = 0.011, respectively; Fig. 8A), similar to taxonomic and broad-scale functional assignments (Fig. 2 and 4). Nine of the most abundant AMR gene families were noted as correlates of the observed differences (Fig. 8B). *VanW* and *vanY* AMR gene families were most abundant in samples collected after 26 weeks (in the austral summer). Higher abundances of sulfonamide resistance, macrolide phosphotransferase (MPH), ANT(3’’), and msr-type ABC-F encoding genes were present in 6-week-old samples. The abundance of the major facilitator superfamily (MFS) antibiotic efflux pump and resistance nodulation cell division (RND) gene family was greatest in two-week-old samples (Fig. 8B). Additionally, it should be noted that the rifamycin-resistant beta subunit of RNA polymerase (*rpoB*) gene and the OXA beta-lactamase gene family showed the highest relative abundance in pond water samples. One of the three pond water replicates contained an unusually high abundance of OXA beta-lactamase sequences. In contrast, the remaining pond water was more strongly characterized by a high abundance of *rpoB*. However, most AMR gene families identified were only represented by a small number of genes, except for ANT(3”) and OXA beta-lactamase gene families (Fig. S6), which have many individual genes contributing to their dominance (Fig. S6).

An age and depth effect can be observed in changes in abundance of the top 1% most abundant AMR gene families (Fig. 8C and D). Relative abundances of ANT (3’’), MPH, msr-type ABC-F protein, and sulfonamide resistance genes were higher at 6 weeks. Conversely, relative abundances of genes encoding for OXA beta-lactamase, RND, and rifamycin-resistance via the beta-subunit of RNA polymerase (rpoB) were greater at 2 and 26 weeks (Fig. 8C). Interestingly, the *vanY* glycopeptide resistance gene was only abundant at 26 weeks. Additionally, the 52 week biofilms had a lower overall abundance of the top 1% most abundant AMR gene families, which were also present at the other sample times (Fig. 8C). The top-tier biofilms contained the most abundant AMR gene families (Fig. 8D). Overall, the top tier was more abundant in ANT(3’’), MPH, msr-type ABC-F protein, and sulfonamide resistance genes than other depths (Fig. 8D). These were all present in the top 1% of AMR gene families in the middle tier, but at lower abundances than in the top tier, whereas the bottom depth had the least abundant AMR gene families. Pond water samples had a distinct composition of AMR gene families, highly prominent in *rpoB*, RND, amd MFS antibiotic efflux pump-related genes. It should be noted that pond water had distinct AMR gene family abundances compared to the biofilm-associated communities. The differences in gene family abundances may impact the overall distribution of gene families when comparing the biofilm age.

## DISCUSSION

The impacts of plastic pollution on the environment (6–8) and macrobiota have been extensively studied in the last few decades (4, 5). More recent studies have also explored impacts on marine microbial communities (10, 75–77), yet the functional impact plastics have on aquatic microbiota remains poorly understood. We reveal broad-scale taxonomic and functional changes that plastics may cause as novel substrates in WWTP maturation ponds. Typically, these microbial attributes were not distinct among plastic substrate types, but we demonstrate that the age and depth of plastisphere biofilms confer compositional and functional differences, as previously elucidated in marine studies by Oberbeckmann et al. (12), Xu et al. (78), and Wallbank et al. (10). Additionally, we found that, while low in abundance, these biofilm-associated communities host functional elements related to plastic degradation and antibiotic resistance. Understanding the effects prolonged exposure to plastics can have on microbial functioning has important implications for bioremediation and conservation efforts.

### Microbial composition

Microbial community colonization and composition depend on the complex relationships between the physical-chemical properties of a substrate and the impacts of surrounding environmental factors (12), such as light, temperature (79), and dissolved organic content. Plastics with smooth surfaces can be resistant, or slower, to develop biofilms by having limited surface abrasion for microbial attachment. Nonetheless, it has been shown that organic matter rapidly accumulates on plastics following immersion, creating a conditioning film on solid substrates (80). Rough surfaces and hydrophobicity can influence conditioning rates and colonization (81); however, external environmental factors continue to influence the community composition (12). These results suggest that environmental factors are leading contributors to core community composition rather than plastic substrate specificity. Our study indicates that colonization, as well as the age and depth of the biofilms on plastic, have a greater impact on diversity than substrate type (12, 80, 81). Moreover, as light and temperature increased over the austral summer, we saw significant increases in Cyanobacteria across all substrate types. Cyanobacteria can proliferate during increased light and temperature periods, surviving harsh UV light and other environmental stressors (82), subsequently dominating the plastisphere (83). Coinciding with the increase in Cyanobacteria, metazoan relative abundance also increased (e.g., there was an increased abundance of crustaceans, annelids, and rotifers; data not shown). It is likely that the Cyanobacteria, in combination with warmer surface waters, provide more nutrients for metazoan grazers. This could have functional implications relating to increases in nutrient availability, toxins (84), and bioremediation of environmental contaminants (85, 86). This same compositional change was not noted in the eukaryotes, suggesting seasonal variation plays a lesser role in the diversity of eukaryotes. Other studies indicate that plastisphere communities change rapidly in the initial stages of colonization to form generalist communities (10) and provide rafting opportunities for potential pathogens (76, 87), depending on the prevailing environmental conditions (87).

It should be noted that through metagenomic read analysis, we failed to identify a significant proportion of the taxa present, particularly at the genera level. Unassembled metagenomic sequences are prone to prediction and alignment issues due to lower query alignment length (88) and subsequently lack phylogenetic depth, which can be improved through 16 rRNA SSU/LSU assembly (89). However, rare taxa are less likely to be sampled through metagenomic assembly and binning due to a lack of these low-read fragments and their reference genomes. Archaea were among the most poorly described taxa observed in this study, only identifying members of the Euryarchaeota phylum. Despite this, there was an observed increase in the class *methanomicrobia* at the final sampling time. Methanogenic Archaea metabolize organic carbon to carbon dioxide and methane in anoxic environments (90, 91). This suggests that as the biofilms age and accumulate biomass, they create an anoxic environment within the biofilms’ interior. Like most Archaea, this class remains poorly understood functionally and poorly documented within the plastisphere, although a recent study confirmed the presence of archaeal plastic-degrading enzymes (92). Similarly, freshwater microeukaryotes are typically poorly described and often overlooked (93). Further studies of freshwater systems would benefit by capturing microbial diversity using quantitative sequencing, targeting key taxonomic groups of interest, e.g., via 16S and 18S rRNA and ITS (internal transcribed spacer) amplicon sequencing.

### Functional potential

As with community composition, the functional potential of the aquatic microbial communities largely depends on environmental conditions, with little difference in functional potential observed between substrate types. Previous studies report that biodegradable plastics can influence the composition and function of microbial communities (94), whereas non-biodegradable plastics have little impact on community functionality (95). Yet, we found no significant distinction between communities associated with biodegradable (i.e., PLA) vs non-biodegradable plastics (i.e., LLDPE, oxo-LLDPE, PA-6, and PET).

Interestingly, biofilms associated with manganese stearate-supplemented LLDPE (i.e., oxo-LLDPE) contained the highest number of predicted functions that indicate microbial growth on a specific plastic (when pond water samples were removed from the data). These functions encompass a range of general housekeeping genes, meaning that manganese stearate (Mn) supplemented oxo-LLDPE may provide a more tolerable substrate for microbes, resulting in higher overall abundances of housekeeping genes. Abrusci et al. (96) and Konduri et al. (97) suggest that Mn can improve the biodegradability of low-density polyethylene (LDPE) compared to conventional LDPE, yet Mn stearate is also reported as an anti-biofouling agent on superhydrophobic surfaces (98). In a parallel study (Wallbank et al., unpublished data), in which we submerged the same plastics in marine water for one year, the most dramatic physical effects were found for the oxygen-degradable LLDPE, with increased crystallinity, intense surface cracking, and substantial deterioration of its mechanical properties.

### Plastic degradation

The biodegradation of plastics by microbial communities is a promising avenue for bioremediation. Several studies demonstrate elevated levels of putative plastic degrading organisms and their respective enzymes following incubation with plastic, yet studies typically focus on communities in more stable environments, such as soils and sediments (99–101). We noted no variation in the relative abundances of putative plastic degradation-encoding genes among most substrates, with pond water having a higher-than-average concentration of genes that encode for putative plastic-degrading enzymes, compared to biofilm communities. These included genes encoding more general biodegradative enzymes such as esterases, lipases, and monooxygenases, found in *Pseudomonas* sp. (102), *Pseudomonas chlororaphis* (103), and *Acinetobacter johnsonii* (101), reported to degrade a range of plastics, including PET and PLA. However, these genes encode that putative plastic-degrading genes were only found in low-relative abundances compared to other functional genes found in the data set, suggesting that it is unlikely that these genes are efficiently degrading plastics. Interestingly, PA-associated communities contained genes related to plastic-specific biodegradation even though polyamides, such as nylon-6 used in this study, are reported as representing <10% of microplastic fragments detected at the Christchurch WWTP (23). These genes included those encoding nylon hydrolases, nylon oligomer-degrading enzymes, and hydrolases found in *Agromyces* sp. (104) and *Paenarthrobacter ureafaciens* (105), respectively. While neither of these were among the top five most abundant bacterial genera detected, these results indicate that PA biofilms harbored the potential to degrade nylon-based substrates. Additionally, PEG dehydrogenase genes were present but not associated with any particular substrate, indicating that the gene may be more abundant in biofilm communities and not a trait specifically linked to plastic biodegradation.

### Antimicrobial-resistance gene prevalence in plastisphere biofilm communities

Plastics are a surface for biofilm formation and conjugation (106), potentially spreading antimicrobial resistance-conferring genes and pathogenic bacteria through the environment. Transferable genetic elements were ubiquitous across all sample types and depths, with the top-tier biofilms at 6 weeks having the highest abundance of all biofilms samples and a high abundance of conjugative transfer elements found in pond water at 52 weeks. We found little difference in the abundances of AMR genes among substrate types, instead noting a distinction between biofilm ages and depths. Specifically, pond water had a distinct abundance and composition of AMR genes compared with the biofilm communities, with AMR genes differentially abundant in pond water. AMR genes were more abundant in biofilms, which may be so through increased conjugation (106). A few genes or gene families were found exclusively on solid substrates and not in pond water. These included ANT(3’’) (aminoglycoside O-nucleotidyltransferase), sulfonamide resistance (*sul*), and the glycopeptide-resistance gene cluster (*vanW*, *vanY*).

ANT(3’’) enzymes inactivate aminoglycoside antibiotics, commonly prescribed for children with infections caused by Gram-negative bacterial pathogens (107). While there appears to be little information regarding this gene family’s abundance in wastewater, these enzymes are typically encoded on mobile genetic elements (108), which would help explain their abundance within biofilms. The *sul* genes encode forms of dihydropteroate that confer resistance to sulfonamide, a class of synthetic antimicrobial drugs that are pharmacologically used as a broad-spectrum treatment of human and animal bacterial infection (109, 110). Sulfonamide resistance is commonly found in wastewater systems (111, 112) and is associated with extreme fecal contamination and poor influent water quality (113). This does not necessarily indicate that the Christchurch municipal WWTP has fecal contamination post-treatment; residual DNA may be a lingering artifact of the treatment process. *VanW* and *vanY* AMR gene families encode proteins involved in glycopeptide antibiotic resistance. *VanW* genes are accessory genes that encode unknown functions found on vancomycin resistance operons, whereas *vanY* encodes for the modification of peptidoglycan terminal subunits to reduce their binding affinity with vancomycin (114, 115). Vancomycin-resistance-conferring genes are often found in wastewater systems (116), commonly associated with *Enterococcus* species (117–119). Finally, there was a substantial increase in the abundance of msr-type ABC-F encoding genes across the shallower, top-tier biofilms. The msr-type ABC-F encoding protein, AMR gene family, is typically expressed in *Staphylococci* species and confers resistance to erythromycin and streptogramin B antibiotics (120). The differential and elevated abundances of these AMR gene families on substrates compared to pond water suggest that these artificial substrates act as platforms for the transmission of AMR, or that the genera with AMR gene families are more likely to increase in relative abundance on solid substrates compared to pond water.

### Conclusion

As plastic pollution persists in our environment, the long-standing effects on freshwater and marine microbiota are an important research area. We provide new insights into the functional potential of plastisphere biofilms in comparison with ambient pond water microbiota. While this study finds little evidence of plastisphere-specific community traits and diversity, we identify evidence that generalist biofilm communities have at least some capacity to interact with and perhaps even degrade plastic polymers and associated substances, which are consistent with the low-energy yields expected from plastic degradation. Additionally, while we found no evidence of increased mobile genetic elements within plastisphere samples, we found evidence of plastics acting as vehicles for establishing and transporting harmful traits, such as AMR. These traits, compounded with high volumes of plastic particles detected in WWTP effluent, are a key concern for the health of macroorganisms and the environment. Overall, this study provides insight into the potential effects plastic waste can have on WWTP microbiota before their release into the wider environment.

## Data Availability

Raw metagenomic sequence data that support the findings of this study have been deposited to the National Center for Biotechnology Information (NCBI) with the primary BioProject accession number PRJNA1134258.

## SUPPLEMENTAL MATERIAL

Supplemental material aem.00715-24-s0001.docx Tables S1 to S5; Figures S1 to S6.

ASM does not own the copyrights to Supplemental Material that may be linked to, or accessed through, an article. The authors have granted ASM a non-exclusive, world-wide license to publish the Supplemental Material files. Please contact the corresponding author directly for reuse.
