# Supplementary material for: Taxonomic variation, plastic degradation, and antibiotic resistance traits of plastisphere communities in the maturation pond of a wastewater treatment plant
Source: Appl Environ Microbiol. 2024 Sep 27;90(10):e00715-24. doi: 10.1128/aem.00715-24 (PMC11497791; doi:10.1128/aem.00715-24)
Supplement: Supplemental material — Tables S1 to S5; Figures S1 to S6. [file aem.00715-24-s0001.docx]

**Supplementary material**

**Table S1** Light (average lux and minutes per day) and temperature (°C) of the wastewater treatment plant study site recorded by HOBO loggers at three depths below the water surface (20, 40 and 60 cm). Data were averaged across these depths for three days after sampling, when HOBO loggers were cleaned. Any spurious readings were removed from the dataset before averaging.

| Sample time | Logger ID | Temp (^o^C) | Lux | Average minutes of light per day |
| --- | --- | --- | --- | --- |
| Deployment (30 July 2020) | Top | 8.85 | 3835 | 635 |
|  | Middle | 8.64 | 1641 | 620 |
|  | Bottom | 8.74 | 1183 | 625 |
| Two weeks (15 August 2020) | Top | 10.39 | 3315 | 675 |
|  | Middle | 10.15 | 1680 | 665 |
|  | Bottom | 10.22 | 656 | 655 |
| Six weeks (12 September 2020) | Top | 12.18 | 3126 | 745 |
|  | Middle | 11.91 | 1124 | 740 |
|  | Bottom | 11.99 | 488 | 730 |
| Six months (31 January 2021) | Top | 19.17 | 4590 | 915 |
|  | Middle | 18.78 | 1205 | 910 |
|  | Bottom | 18.77 | 314 | 895 |
| 12 months* (26 July 2021) | Top | 9.54 | 4381 | 630 |
|  | Middle | 9.42 | 1274 | 630 |
|  | Bottom | 9.53 | 517 | 615 |

*averages were taken for the three days before sampling due to dataloggers being removed at the conclusion of sampling.

**Table S2** Polymer type and additive composition used in the experimental design, as described by suppliers.

| **Plastic Type** | **Base Polymer** | **Known additive** | **Additive content** |
| --- | --- | --- | --- |
| Linear low-density polyethylene (LLDPE) | Innoplus LL7410D | *Irganox B215 (33% Irganox 1010 and 67% Irgafos 168) | 0.25% |
| Oxygen-degradable LLDPE (oxo-LLDPE) | Innoplus LL7410D | *Manganese stearate | 0.20% |
| Polyamide 6 (PA) | Ultramid B3S | Nylostab S-EED  Talc | 0.50% |
| Polyethylene terephthalate (PET) | PAPET COOL IV 0.80 | Tinuvin 234 | 0.30% |
| Polylactic acid (PLA) | Ingeo Biopolymer 3052D | Ethylene bis(stearamide) | < 2.0% |

*the additive AO-1076 are presumed to have been incorporated into the virgin polyethylene resin at 0.13 wt% concentration (Bridson et al., submitted).

**Table S3** Inorganic content of study plastics as detected by thermogravimetric analysis. Mean residual masses are presented as percentages and absolute weights (mg).

|  | | PLA | oxo-LLDPE | LLDPE | PA | PET |  |
| --- | --- | --- | --- | --- | --- | --- | --- |
| Initial mass (mg) | Average | 18.700 | 11.436 | 21.864 | 28.754 | 16.263 | |
|  | Stdev | 5.573 | 2.027 | 3.016 | 7.778 | 4.764 | |
| Residual mass (%) | Average | -0.106 | 0.180 | 0.258 | 0.200 | 0.031 | |
|  | Stdev | 0.084 | 0.074 | 0.066 | 0.034 | 0.136 | |
| Residual mass (mg) | Average | -0.018 | 0.019 | 0.056 | 0.057 | 0.011 | |
|  | Stdev | 0.013 | 0.006 | 0.017 | 0.019 | 0.020 | |

**Table S4** Average trace element concentration (mg/kg) within plastic paddles Data are highlighted in orange when there were significant changes over time as per single factor ANOVA (P < 0.05). Data are highlighted in pale orange when there is a non-significant change.

|  | Detection limits (mg/kg) | LLDPE | | | oxoLLDPE | | | PET | | | PLA | | | PA | | |
| --- | --- | --- | --- | --- | --- | --- | --- | --- | --- | --- | --- | --- | --- | --- | --- | --- |
| Time |  | t0 | t26 | t52 | t0 | t26 | t52 | t0 | t26 | t52 | t0 | t26 | t52 | t0 | t26 | t52 |
| Al | 7.5 | 95.8 | 104.1 | 95.7 | 87.0 | 102.0 | 76.0 | 12.4 | 35.8 | 15.9 | 15.2 | 42.3 | 8.3 | 43.2 | 49.1 | 27.0 |
| P | 0.8 | 87.4 | 146.0 | 141.0 | 46.4 | 113.3 | 118.8 | 16.4 | 31.4 | 38.9 | 0.2 | 8.3 | 14.8 | 1.1 | 42.0 | 31.0 |
| Cr | 0.1 | ND | 0.2 | 0.2 | ND | 0.3 | 0.3 | 0.2 | 0.2 | 0.2 | ND | 0.2 | 0.1 | 0.1 | 0.2 | 0.3 |
| Mn | 0.8 | 0.1 | 7.5 | 7.7 | 91.0 | 126.5 | 123.0 | ND | 8.8 | 5.5 | ND | 7.0 | 5.9 | 0.1 | 7.6 | 8.4 |
| Fe | 7.5 | 9.6 | 9.5 | 25.8 | 3.9 | 12.6 | 35.0 | 2.2 | 8.8 | 12.2 | 5.1 | 13.9 | 13.5 | 9.0 | 9.2 | 24.8 |
| Co | 0.1 | ND | ND | ND | ND | ND | ND | 21.7 | 21.4 | 22.5 | ND | ND | ND | ND | ND | ND |
| Ni | 0.8 | ND | 0.1 | ND | ND | 0.1 | 0.3 | 0.1 | 0.2 | 0.1 | ND | 0.1 | ND | ND | ND | ND |
| Cu | 4 | ND | 0.2 | 0.1 | 6.5 | 0.3 | 0.2 | ND | 0.1 | ND | ND | 0.1 | ND | ND | 0.1 | ND |
| Zn | 4 | 683.2 | 699.5 | 698.9 | 660.5 | 724.4 | 646.9 | ND | 0.5 | ND | ND | 0.6 | ND | ND | ND | 0.3 |
| Sr | 0.8 | ND | 0.4 | 0.1 | ND | 0.2 | 0.2 | ND | 0.1 | ND | ND | 0.1 | ND | 0.1 | 0.5 | 0.1 |
| Pb | 0.8 | ND | 0.1 | 0.1 | ND | 0.1 | 0.1 | ND | ND | ND | 0.1 | ND | ND | ND | ND | ND |

*ND – not detectable using current methods

**Table S5** Trace element concentrations (mg/kg) in plastic-associated biofilms. Data highlighted in red are arranged by minimum to maximum, maximum being dark red and minimum being white.

|  | Detection limit (mg/kg) | LLDPE-A | LLDPE-A | PA-A | PA-A | PET-A | PET-A | PLA-A | PLA-A | oxo-LLDPE-A | oxo-LLDPE-A |
| --- | --- | --- | --- | --- | --- | --- | --- | --- | --- | --- | --- |
| Timepoint | | 26 | 52 | 26 | 52 | 26 | 52 | 26 | 52 | 26 | 52 |
| Al | 7.5 | 7955.3 | 9525.5 | 6143.4 | 11297.5 | 8706.4 | 9837.9 | 10696.1 | 10369.3 | 6697.3 | 9790.0 |
| P | 0.8 | 9224.1 | 12264.3 | 7830.1 | 10473.5 | 6463.0 | 11619.6 | 6451.0 | 9434.3 | 6464.6 | 11176.4 |
| Cr | 0.1 | 155.1 | 120.3 | 181.9 | 137.0 | 251.3 | 142.4 | 229.4 | 112.5 | 152.3 | 152.6 |
| Mn | 0.8 | 1593.2 | 3747.7 | 506.9 | 3129.8 | 542.8 | 3540.6 | 745.2 | 3068.3 | 845.3 | 3178.8 |
| Fe | 7.5 | 8341.7 | 16164.2 | 6722.4 | 16914.9 | 10136.2 | 15917.5 | 10939.9 | 15946.5 | 7223.0 | 16370.8 |
| Co | 0.1 | 3.4 | 4.2 | 2.9 | 4.5 | 5.1 | 4.5 | 4.5 | 4.4 | 2.9 | 4.5 |
| Ni | 0.8 | 39.5 | 20.0 | 53.6 | 19.9 | 84.0 | 20.6 | 72.1 | 19.2 | 40.7 | 21.6 |
| Cu | 4 | 93.6 | 85.9 | 66.1 | 76.9 | 65.9 | 83.3 | 67.9 | 65.0 | 62.9 | 86.3 |
| Zn | 4 | 244.1 | 272.3 | 224.2 | 267.6 | 232.0 | 275.7 | 238.5 | 241.9 | 368.2 | 311.2 |
| As | 4 | 3.8 | 6.3 | 3.9 | 7.6 | <4 | 5.6 | <4 | 5.5 | <4 | 5.7 |
| Sr | 0.8 | 70.6 | 93.0 | 40.3 | 87.2 | 47.9 | 88.0 | 52.5 | 78.3 | 45.1 | 88.3 |
| Zr | 7.5 | 0.0 | 0.0 | 0.0 | 0.0 | 0.0 | 0.0 | 0.0 | 0.0 | 0.0 | 0.0 |
| Mo | 0.8 | 3.3 | 2.4 | 2.3 | 2.2 | 3.3 | 2.2 | 2.4 | 2.3 | 2.3 | 2.9 |
| Ru | 0.1 | 0.0 | 0.0 | 0.0 | 0.0 | 0.0 | 0.0 | 0.0 | 0.0 | 0.0 | 0.0 |
| Cd | 0.1 | 0.0 | 0.0 | 0.0 | 0.0 | 0.0 | 0.0 | 0.0 | 0.0 | 0.0 | 0.0 |
| Cs | 0.8 | 1.3 | 1.4 | 1.0 | 1.8 | 1.4 | 1.5 | 1.7 | 1.6 | 1.1 | 1.5 |
| Ba | 0.8 | 98.7 | 152.1 | 54.1 | 147.0 | 63.3 | 149.2 | 78.7 | 128.3 | 68.3 | 148.3 |
| Ce | 0.1 | 14.4 | 21.7 | 10.9 | 26.9 | 23.6 | 19.5 | 25.8 | 26.1 | 14.8 | 23.4 |
| Hg | 0.1 | 0.2 | 0.2 | 0.3 | 0.3 | 0.2 | 0.3 | 0.3 | 0.2 | 0.2 | 0.3 |
| Pb | 0.8 | 25.2 | 29.9 | 15.2 | 31.3 | 18.2 | 31.4 | 22.6 | 26.6 | 16.3 | 31.0 |


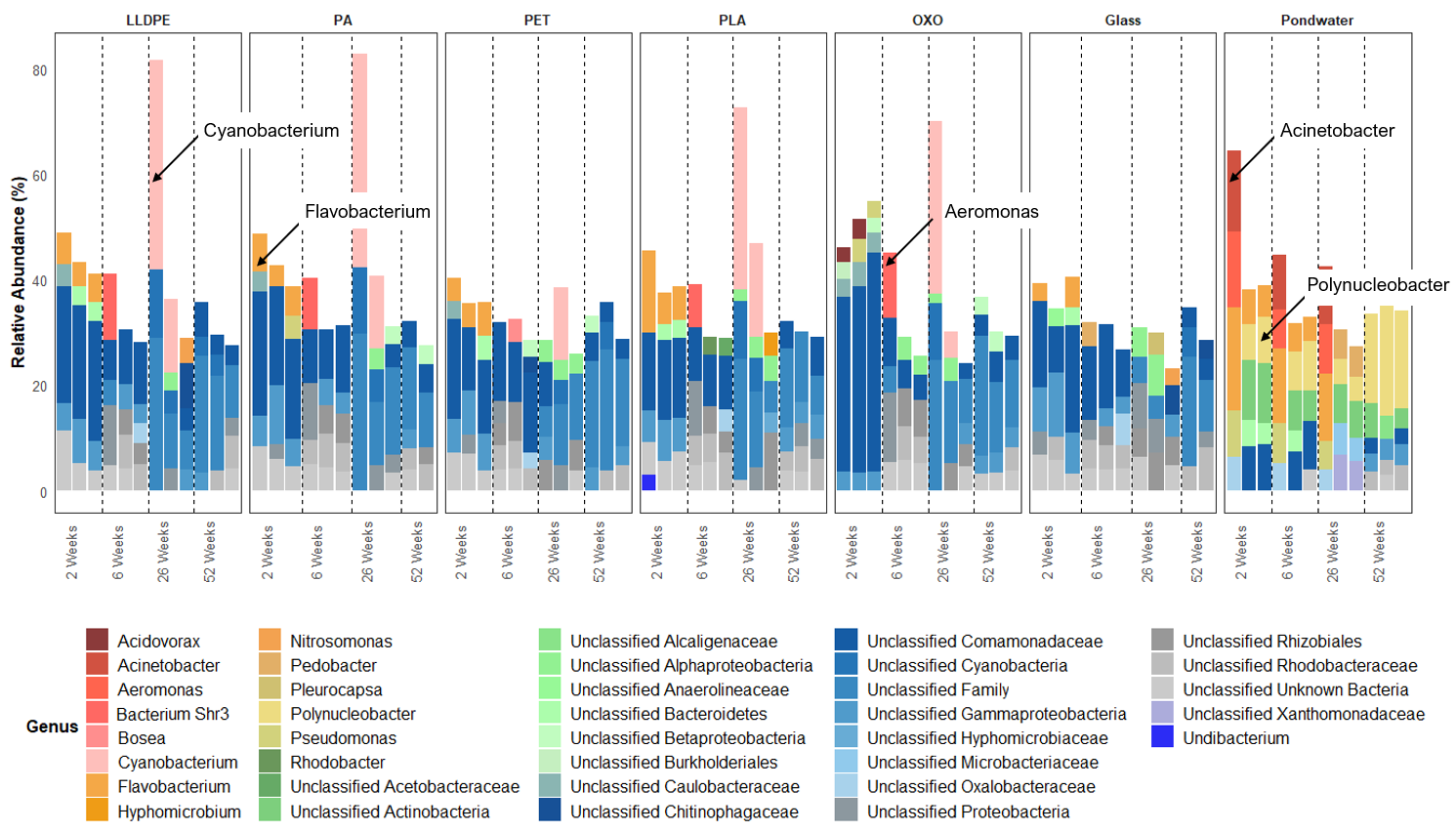


**Figure S1** Top five most abundant bacterial genera for each substrate, including within the maturation pond water. Dashed lines separate the different ages of the biofilm samples, organised as Top, Middle, and Bottom (left to right) within each age group. Substrates included linear low-density polyethylene (LLDPE), oxygen-degradable LLDPE (OXO), nylon-6 (PA), polyethylene terephthalate (PET), polylactic acid (PLA) and glass as a control.


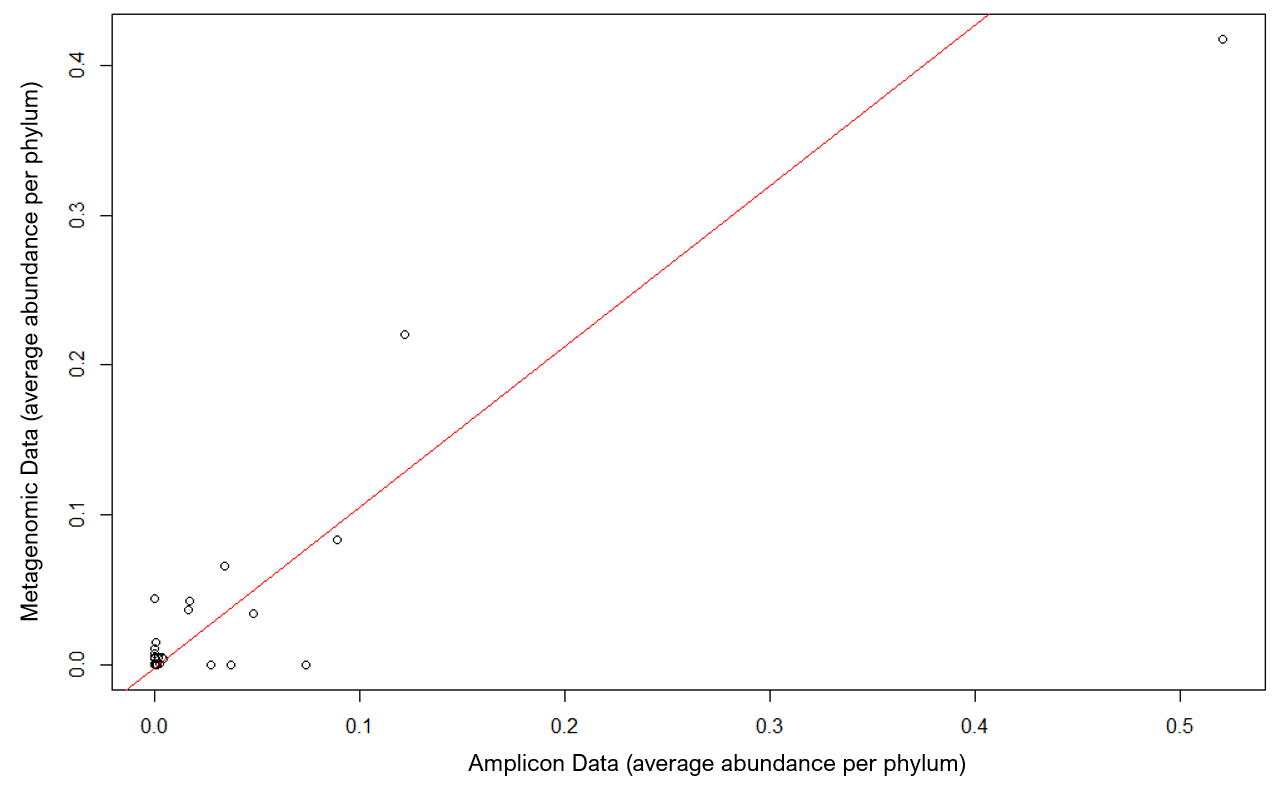


**Figure S2** Correlation of unassembled metagenomic read taxonomic assignments and 16S rRNA amplicon taxonomic assignments (Wallbank et al., unpublished data) to phylum level (P <0.01, F = 86.88, R2 = 0.984, 10 df). Unassembled reads were assigned based on metaxa default databases (Bengtsson‐Palme et al., 2015) and converted to relative abundance data. 16S rRNA gene regions were assigned to taxa using the DADA2 pipeline and a Silva database. The red line indicates the path of a perfect (1:1) correlation.


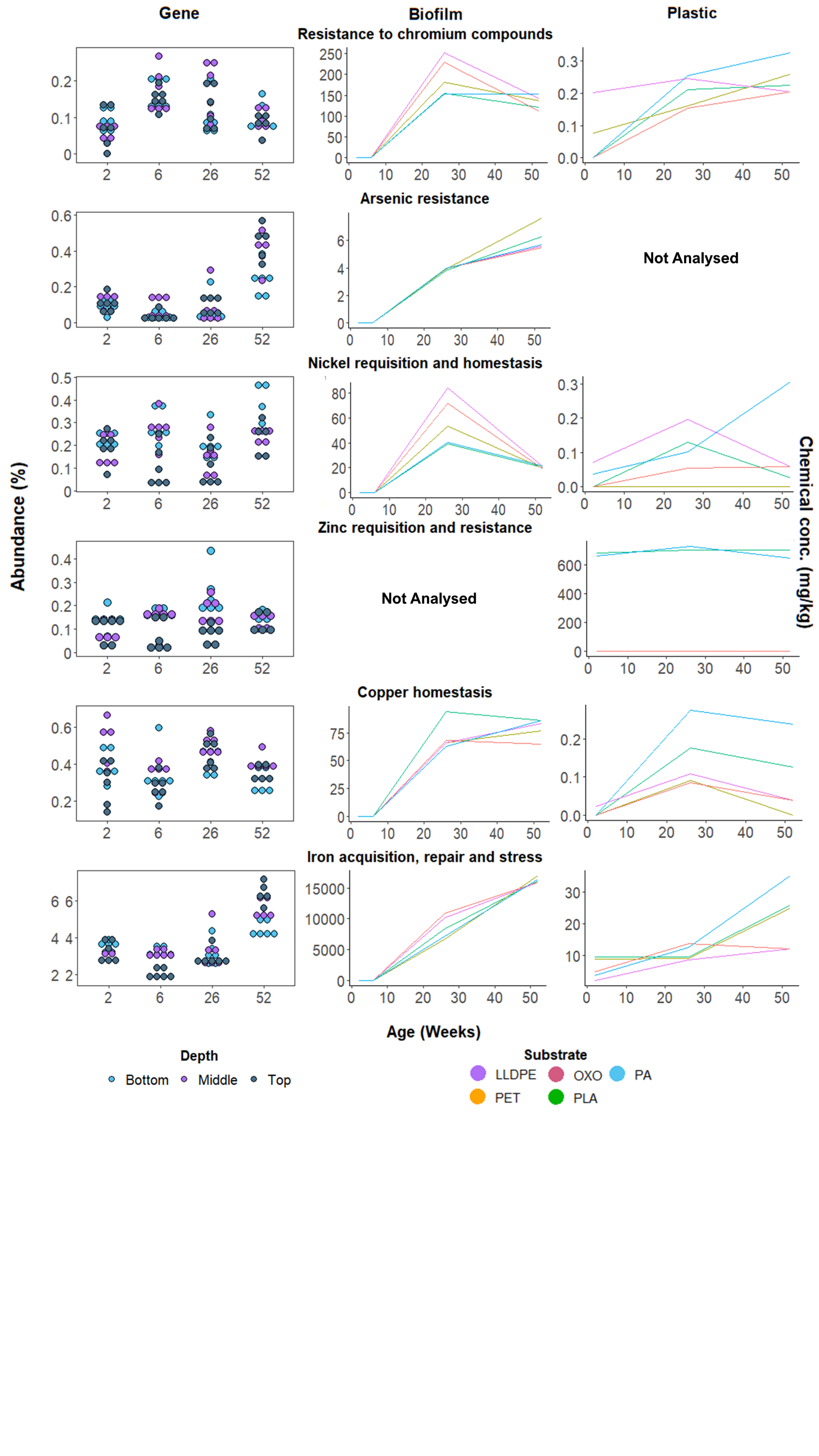


**Figure S3** Relative abundance (%) of functions associated with metals found in plastic paddles and plastic-associated biofilms as assigned by a SEED hierarchy, organised by depth from the surface of the maturation pond water: Top (20 cm from the surface), Middle (40 cm from the surface), and Bottom (60 cm from the surface). Note that zinc was not tested in biofilms, and arsenic was not tested in plastic paddles. All other metals found in plastic and plastic-associated biofilms did not have SEED assignments directly relative to homeostasis, stress responses or resistance.


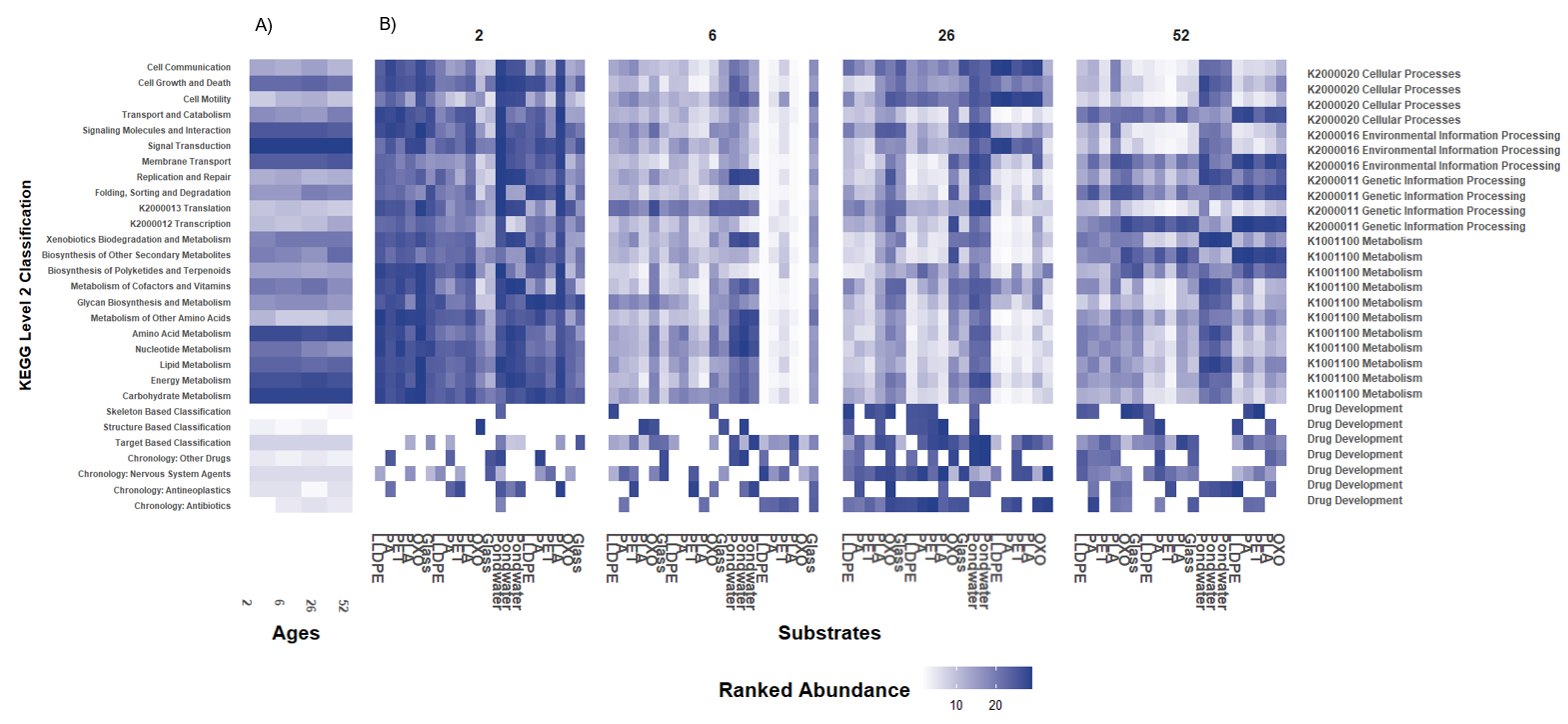


**Figure S4** Abundance of cumulative sum non-assembled shot metagenomic first (right axis) and second level (left axis) Kyoto Encyclopaedia of Genes and Genomes (KEGG) functions. Predicted coding regions used in KEGG diamond blast originate from glass and plastic-associated biofilms. Substrates include glass, linear low-density polyethylene (LLDPE), polyamide (PA)), polyethylene terephthalate (PET), polylactic acid (PLA), oxygen-degradable LLDPE (oxo-LLDPE) and a filtered pond water control. (A) Sequentially adjusted abundances highlight which samples contain the most genes relevant to each KEGG category. (B) Relative abundance of KEGG sequences.


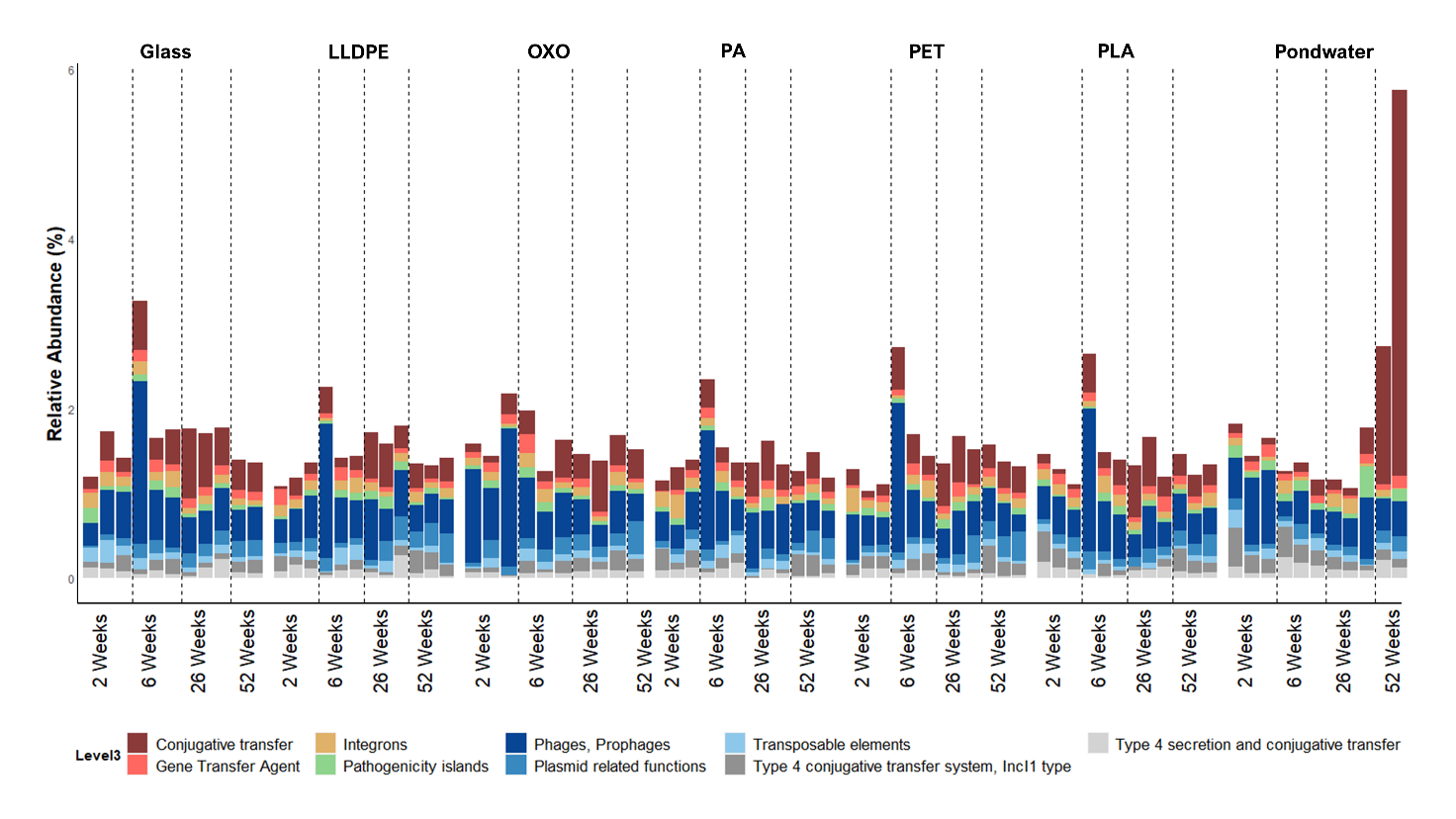


**Figure S5** Relative abundance of transferrable genetic elements. Dashed lines separate the different ages of the biofilm samples, which, within these age groupings, are organised as Top, Middle, and Bottom (left to right). Substrates included linear low-density polyethylene (LLDPE), oxygen-degradable LLDPE (OXO), nylon-6 (PA), polyethylene terephthalate (PET), polylactic acid (PLA), and glass as a control.


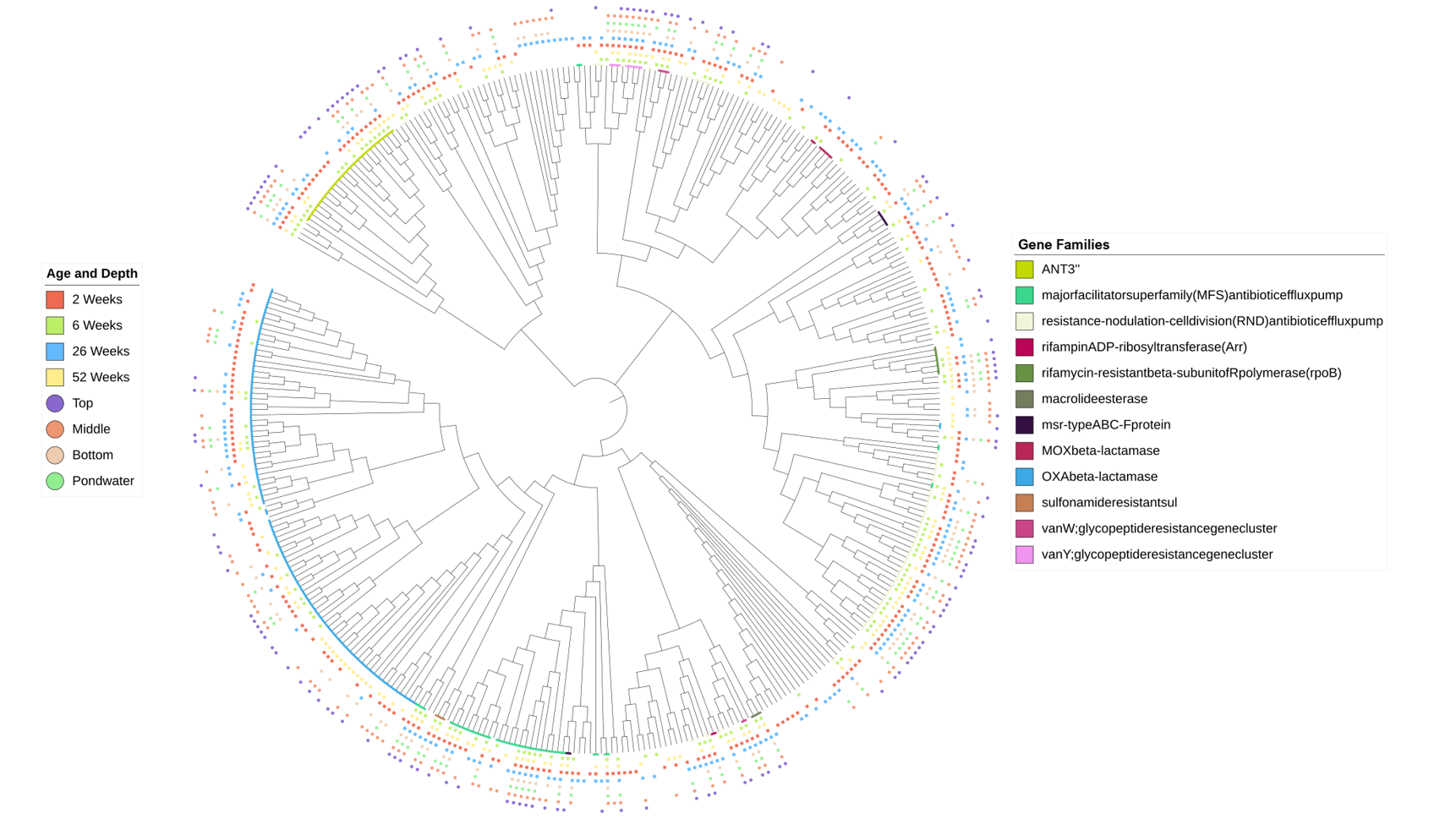


**Figure S6** Maximum likelihood phylogenetic tree of antimicrobial resistance (AMR) genes found in wastewater treatment plant biofilms, based on Le and Gascuel models of amino acid evolution (Le and Gascuel, 2008). Genes are grouped by the most abundant gene family and the biofilm sample at either depth or age. Innermost label represents the gene families, middle labels (coloured squares lime green - blue) indicate the age of the biofilms AMR genes were present, and the outer most labels (coloured circles beige – purple) indicate the depth of the biofilms.
